# Quantum Dots Assembled with Photosynthetic Antennae on a Carbon Nanotube Platform: A Nanohybrid for the Enhancement of Light Energy Harvesting

**DOI:** 10.1021/acsomega.3c07673

**Published:** 2023-10-26

**Authors:** Jakub Sławski, Jan Maciejewski, Rafał Szukiewicz, Katarzyna Gieczewska, Joanna Grzyb

**Affiliations:** † Department of Biophysics, Faculty of Biotechnology, University of Wrocław, F. Joliot-Curie 14a, 50-383 Wrocław, Poland; ‡ Faculty of Physics and Astronomy, University of Wrocław, Maxa Borna 9, 50-204 Wrocław, Poland; § Department of Plant Anatomy and Cytology, Institute of Experimental Plant Biology and Biotechnology, Faculty of Biology, University of Warsaw, I. Miecznikowa 1, 02-096 Warsaw, Poland

## Abstract

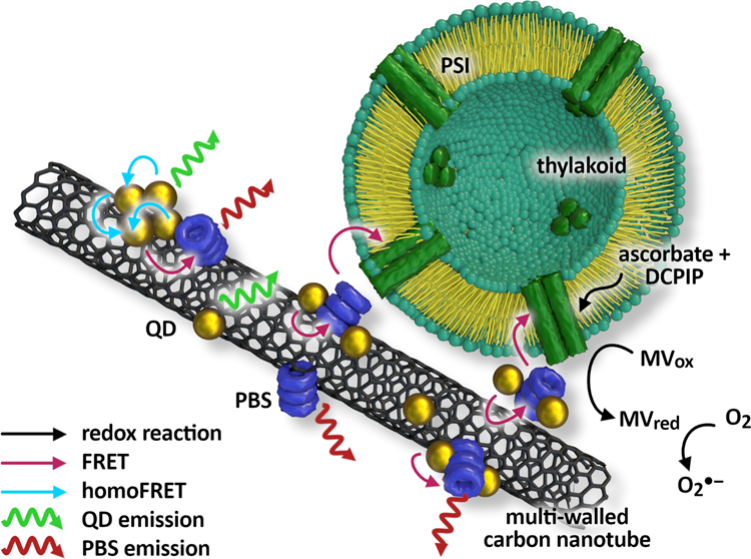

The construction of artificial systems for solar energy harvesting is still a challenge. There needs to be a light-harvesting antenna with a broad absorption spectrum and then the possibility to transfer harvested energy to the reaction center, converting photons into a storable form of energy. Bioinspired and bioderivative elements may help in achieving this aim. Here, we present an option for light harvesting: a nanobiohybrid of colloidal, semiconductor quantum dots (QDs) and natural photosynthetic antennae assembled on the surface of a carbon nanotube. For that, we used QDs of cadmium telluride and cyanobacterial phycobilisome rods (PBSr) or light-harvesting complex II (LHCII) of higher plants. For this nanobiohybrid, we confirmed composition and organization using infrared spectroscopy, X-ray photoelectron spectroscopy, and high-resolution confocal microscopy. Then, we proved that within such an assembly, there is a resonance energy transfer from QD to PBSr or LHCII. When such a nanobiohybrid was further combined with thylakoids, the energy was transferred to photosynthetic reaction centers and efficiently powered the photosystem I reaction center. The presented construct is proof of a general concept, combining interacting elements on a platform of a nanotube, allowing further variation within assembled elements.

## Introduction

Colloidal quantum dots (QDs) are semiconductor nanocrystals known for their luminescence properties and the ability to transfer energy by resonance mechanisms as well as to donate photoinduced electrons to various acceptors or electrodes.^1−3^ QDs are usually quasispheres of a few nanometers in diameter. In this research, we used core-only QDs composed of CdTe. The surface of these QDs is passivated by carboxythiols, resulting in a negative charge of the nanoparticle in the majority of water-based solutions. The carboxyl group might also be activated and used for cross-linking with proteins or other molecules containing primary amine moieties to form the nanohybrid composite of different elements. In such connections, their final function is defined by the particular components; it might be the function of QD-based antennae for further energy acceptors as well as the QD-based powering of enzymatic reactions.

Photosynthetic antennae are pigment–protein complexes auxiliary to photosystems constituting the photosynthetic reaction centers. The antennae harvest light energy, enhancing the overall absorption yield by simply increasing the absorption-prone area. Therefore, significantly more excitons reach photosystems, and more light energy is stored into assimilates. Chloroplasts of higher plants and green algae contain two types of antennae, namely, light-harvesting complex I (LHCI) and light-harvesting complex II (LHCII). Both are huge transmembranal protein complexes, assembling chlorophyll and carotenoid molecules. The structure of LHCs ensures close contact between chlorophyll molecules, assuring the productive use of excitation. The minimal model of LHCs, the so-called protein maquette, consists of a four-helix bundle binding porphyrin molecules in its center. Examples are HP7 and RCM proteins.^4,5^ Such proteins are easily expressed and purified from the heterologous system and might be assembled with cofactors to play the role of antennae in vitro. We used one of those to exploit the possibility of their application in the nanobiohybrids. LHCs are absent in the photosynthetic membranes of cyanobacteria; instead, phycobilisomes (PBS) work as antennae.^6^ PBS is very soluble in water. Their attachment to the membrane has a peripheral character and is formed by specialized connector proteins. The structure of PBS consists of phycocyanin monomers that oligomerize to form rods. The chromophores here are phycocyanobilines, present in three copies per functional phycocyanin unit. The absorbed light might form an exciton to a given extent, traveling within such rods to reach the sink in a photosystem. Photosynthetic proteins are often considered a part of nanobiodevices, working as biosensors or alternative energy sources.^7^ In one of the noteworthy examples, Z-scheme mimics were constructed using a photoanode of photosystem II-PbS quantum dots assembled on a TiO_2_ electrode.^8^ In this approach, a bilirubin oxidized modified opal-ATO plate served as a cathode.

Carbon nanotubes (CNTs) are examples of carbon-based nanomaterials. Basically, they are graphene fragments bent in the shape of a tube. Chemically, they are pure carbon, with atoms arranged in a hexagonal pattern. The diameter of tubes varies depending on the number of walls,^9^ from a few nanometers for single-walled CNTs (SWCNTs) to dozens of nanometers for multiwalled CNTs (MWCNTs). The total length of a tube may reach several micrometers. CNTs are good electrical and thermal conductors,^10^ which makes their use in nanodevices attractive.^11^ Multiple approaches have already been presented in the field, with pioneering works published at the turn of centuries, from groups of Lisdat,^12^ Dekker,^13^ Hibbert,^14^ Rusling,^15^ and others. CNTs were shown even to serve as an excellent transducer in complex systems, such as spinach thylakoids and electrode surfaces.^16^ Added to an electrode surface, they significantly increased the electrochemically active area. The variation of this application was a mixture of CNTs with isolated enzymes or even a fruit pulp.^17,18^ CNTs might be decorated with proteins (as enzymes,^19^) and pigment–protein complexes, as well as with other nanomaterials or simpler chemicals.^20,21^ For this purpose, they need to be activated by oxidation or other processes, forming reactive sites on their surface. CNTs might be solubilized by peptides in a diameter-selective manner.^22^ CNTs are known to be able to penetrate the cells of both animals and plants.^23,24^ In plants, it was shown that CNTs might traverse a chloroplast membrane, and the CNT’s surface may be partially covered by a thylakoid membrane.^23,25^ Covalent conjugation of the photosynthetic reaction center, photosystem I (PSI), with CNTs^26−29^ or graphene (being a CNT unrolled surface)^29,30^ has been created, providing potential optoelectronic applications. In this system, researchers observed efficient energy and electron transfer from PSI to CNTs, showing that a nanotube may serve as a final electron sink. Similar results were obtained for another example of photosynthetic complexes, peridinin–chlorophyll–protein, in assembly with CNTs.^31^

Bionanohybrids, defined as a composite of nanomaterials with biological molecules, offer unique features for multiple applications. They might be used for creating constructs that work as elements of green energy production. The main aim of this work is to demonstrate carbon nanotubes as a platform that connects quantum dots and phycobilisome rods (PBSr) or LHCII in a controlled manner. To prove the connection, we chose to observe resonance energy transfer between QDs and PBSr or LHCII. Such a system has a broad absorption spectrum, and we demonstrate that it can be included in longer energy transfer chains, using native cyanobacterial thylakoids with photosynthetic machinery as the final energy acceptors”.

The first advantage of using CNTs in such a system is simply bringing together QDs and photosynthetic antennae. A similar idea was already explored in studies with PSI bridged with PbS QDs^8^ or a cytochrome c molecule.^29^ For example, it is expected that outside of the space of close mutual proximity, there is a very low chance for energy transfer between QDs and PBSr/LHCII. For energy transfer to occur, QDs and PBS need to be connected covalently^32^ or assembled into electrostatically associated clusters.^33^ The assembly studied here of QDs and PBSr/LHCII on a nanotube is a form of covalent connection that offers more specific options for the complex architecture. To some extent, the stoichiometry as well as organization of nanohybrid components may be controlled. Finally, the sequential assembly may result in monolayers coating a tube, while simultaneous conjugation may result in a homogeneous distribution of particles on the CNT surface. Hence, QDs work as additional antennae for PBSr/LHCII. The absorption of the created composite covers the whole visible light range. This significantly increases the utilization of incident light. However, to be really useful, absorbed light needs to be put to work: to show that the downhill energy transfer from PBS to photosystems was assayed, to check if the energy absorbed by QDs and transferred via PBS might power the photosynthetic reactions, and that such nanohybrids might be treated as green building elements for energy-harvesting devices.

## Materials and Methods

### Chemicals

MWCNT, SWCNT, HEPES, bovine serum albumin (BSA), *N*-(3-dimethyl aminopropyl)-*N*′-ethyl carbodiimide (EDC), and *N*-hydroxysuccinimide (NHS) were purchased from Merck, Germany. QDs were obtained from PlasmaChem GmbH, Germany. Luria-Broth medium was purchased from BTL Polska. LHCII was isolated from local market spinach by a method described in ref (34). PBSr was isolated from *Synechocystis* PCC 6803 by the method described in ref (33).

Thylakoids were isolated from *Synechocystis* PCC 6803. The cyanobacterial cells were collected by centrifugation (3000*g*, 10 min) and resuspended in thylakoid buffer (50 mM HEPES/NaOH, pH 7.3, 5 mM MgCl_2_, 10 mM CaCl_2_, 5% (v/v) glycerol). The cell suspension was homogenized using an LM20 Microfluidizer (IDEX Health & Science, WA), with three cycles under 15,000 psi pressure. The homogenate was centrifuged (3000*g*, 10 min) to remove unbroken cells and debris. Then, the thylakoid membranes were pelleted by centrifugation (23,000*g*, 20 min): the dark blue supernatant containing PBSr was removed, and the pellet was washed with thylakoid buffer. The total chlorophyll concentration in the final suspension of thylakoids was determined according to ref (35) and was 360 μg/mL.

A photosynthetic model antenna, HP7 protein, was expressed in *Escherichia coli* and purified by the method described in ref (4). Its cofactor, Zn-mesoporphyrin, was purchased from Frontier Scientific Inc. (DE). All other chemicals came from Carl-Roth GmbH, Germany, and were of “purity for analysis” or higher quality.

### Nanohybrid Preparation

Commercially available MWCNTs were washed with chloroform; ca. 100 mg of CNTs was suspended in a solvent, sonicated for 10 min until the suspension was clear and homogeneous, and centrifuged (10 min, 2000*g*). The procedure was repeated on the pellet two more times. Finally, CNTs were dried and transferred to excess piranha solution for overnight oxidation. The incubation was performed on ice with constant stirring. After oxidation, the suspension was diluted with water and neutralized with 4 M NaOH. Oxidized CNTs were collected on a paper filter, washed with excess water, and stored as a dried powder. The same procedure was successfully applied to SWCNTs.

For conjugation with BSA, ∼30 mg of CNT was suspended in 1 mL of 25 mM HEPES/NaOH, pH 7.3. BSA was added from its water stock solution to a final concentration of ∼30 mg/mL. If necessary, the suspension was sonicated for 3 min. Finally, 1 mg of EDC and 2 mg of NHS were added to start the reaction. The mixture was incubated for 1 h at 30 °C, followed by centrifugation (5 min, 10,000*g*) and resuspension in water. The procedure was repeated three more times to remove unbound BSA and EDC/NHS excess. Final preparation was suspended in water and stored at 4 °C. This preparation was then used for the following attachment of PBSr or LHCII and QD530 or QD570, respectively. For example, 50 μL of CNT-BSA (∼30 μg/mL) was mixed with 5 μL of 300 μM QD530 and 50 μL of 9 μM PBS or with 5 μL of 300 μM QD570 and 10 μL of LHCII preparation (chlorophyll concentration 55 μg/mL), and the final volume was adjusted to 500 μL with 25 mM HEPES/NaOH, pH 7.3. Then, EDC/NHS was added, and the conjugation was performed for 1 h at 30 °C, followed by washing with water or buffer, depending on the final application of the preparation.

### Absorption and Fluorescence Measurement

Absorption and transmission spectra were measured by using a DU800 spectrophotometer (Beckman Coulter, CL).

Steady-state and time-correlated single photon counting (TCSPC) fluorescence measurements were done using an FS5 spectrofluorometer (Edinburgh Instruments, U.K.) equipped with a thermostated cuvette holder. Typically, 3 nm slits are used for the emission and excitation of light beams. The excitation light source was a xenon lamp for steady-state excitation or a 405 nm laser diode for TCSPC. Emission decays were fitted in dedicated Fluoracle software (Edinburg Instruments, U.K.).

For the simulation of transmittance-only dependent reduction of the fluorescence intensity, the calculation was made assuming *F*_0_ = A for *T* = 100% and the reduction of intensity by reduction of transmittance. For example, for *T* = 50%, *F* = 0.5A, assuming 50% lower light intensity induced the luminescence of the sample with given fluorescence yield, etc. Then, the following *F*_0_/*F* was calculated and plotted versus the MWCNT concentration.

### Fourier-Transform Infrared Spectroscopy (FT-IR)

FT-IR spectra were recorded with a Cary 600 Spectrometer (Agilent, CL) with a diamond attenuated total reflection (ATR) unit. Samples were deposited on the ATR by employing gentle drying with a stream of nitrogen. Before the deposition, nanohybrid samples were transferred to the water. Spectra (30 repetitions, accumulated) were recorded with 1 cm^–1^ resolution and processed with dedicated MicroLab Expert software (Agilent, CL). Additional processing (baseline correction and smoothing) was done using OriginPro software (OriginLab, MS).

### Confocal Microscopy (CLSM)

For CLSM and fluorescence lifetime imaging (FLIM), a drop of nanohybrid sample was deposited between two cover glass slides. Imaging was performed using a Stellaris confocal system (Leica, Germany) with a tunable pulsed white laser. HC PL Apo 100*x*/1.4 oil objective was used. Emission ranges were set with a monochromator. Image collection and FLIM processing were done using LasX software (Leica, Germany), and the detailed analysis of FLIM images was performed using a self-written Python script (see the Supporting Information, section Analysis of FLIM images). Possible tail emissions of QDs at 625–667 and 700–799 nm were measured separately and subtracted.

For high-resolution CLSM, nanohybrid samples were washed with water and dried on glass cover slides. Images were collected using the Elyra7 confocal system (Zeiss, Germany), equipped with an HC PL Apo 100*x*/1.4 oil objective. Samples were partially dried on a coverslip before imaging to ensure no movement of nanohybrid particles during measurement. Fluorescence was excited with laser diodes: 405, 488, or 633 nm, depending on the fluorophore. Emission was collected using band-pass filters (420–480 and 495–590 nm) or broad-range FS filters with dichroic mirrors at 405/488/561/633. SIM^2^ (structured illumination microscopy) processing, increasing resolution to 60 nm, was done using Zen Black (Zeiss, Germany) dedicated software. No additional processing was applied except for contrast adjustment of particular channels for easier reading.

### Atomic Force Microscopy (AFM)

AFM measurements were performed with an Agilent 5500 microscope working in contact mode under the control of PicoView software (Agilent, CL). Samples were imaged in liquid cells, and SNL probes (Bruker Nano Inc., Germany) with nominal *k* = 0.35 N/m were used. Samples were deposited on freshly cleaved mica, pretreated previously with poly-l-lysine (Merck, Germany). Image processing was done using Gwyddion software.^36^

### Oxygen Consumption

Oxygen consumption was measured using a Clark-type oxygen electrode (Hansatech, U.K.), using a Schott KL2700 light-emitting diode (LED) lamp (Schott, Germany) as an illumination source. Optical band-pass filters (Omega Optical, VT) with transmission ranges of 510–530 nm (green) and 650–670 nm (red) were used to control the excitation wavelength range of the illuminating light (1500 μmol m^–2^ s^–1^; the intensity was adjusted according to the lamp light spectrum). The samples (1 mL in thylakoid buffer) were prepared in dim light and contained thylakoids (the amount corresponding to 20 μg/mL chlorophyll) with 1 mM sodium ascorbate +0.1 mM 2,6-dichlorophenolindophenol (DCPIP) as electron donors and 0.5 mM methyl viologen (MV) as an electron acceptor. In MWCNT-containing samples, the concentration of MWCNTs was ∼400 μg/mL. The samples were placed in the electrode chamber, and the measurements were started in the dark at room temperature. After 2 min and stabilization of the signal curve, the light was turned on, and the measurement was continued for the next 5–8 min.

### XPS

X-ray photoelectron spectroscopy (XPS) has been used for the analysis of the surface chemical composition. All measurements were performed using an AES/XPS system EA10 (Leybold-Heraeus GmbH, Cologne, Germany). The non-monochromatized X-ray Mg Kα excitation source was used. The overall resolution of the spectrometer during measurements was 0.96 eV as full width at half-maximum (fwhm) of the Ag 3d5/2 line. During measurements, the pressure was kept at a 10^–9^ mbar range. All acquired spectra were calibrated to adventitious carbon C 1s at 285 eV. After subtraction of the Shirley-type background, the core-level spectra were decomposed into main components with mixed Gaussian–Lorentzian lines (70% G + 30% L for the majority of photopeaks) by a nonlinear least-squares curve-fitting procedure, using CasaXPS software. The atomic concentration was determined based on XPS spectra analysis, taking into account the presence of individual elements O, C, Cd, N, S, Te, Cu, and Fe.

## Results

### CNTs as Fluorescence Quenchers

**Figure 1:**
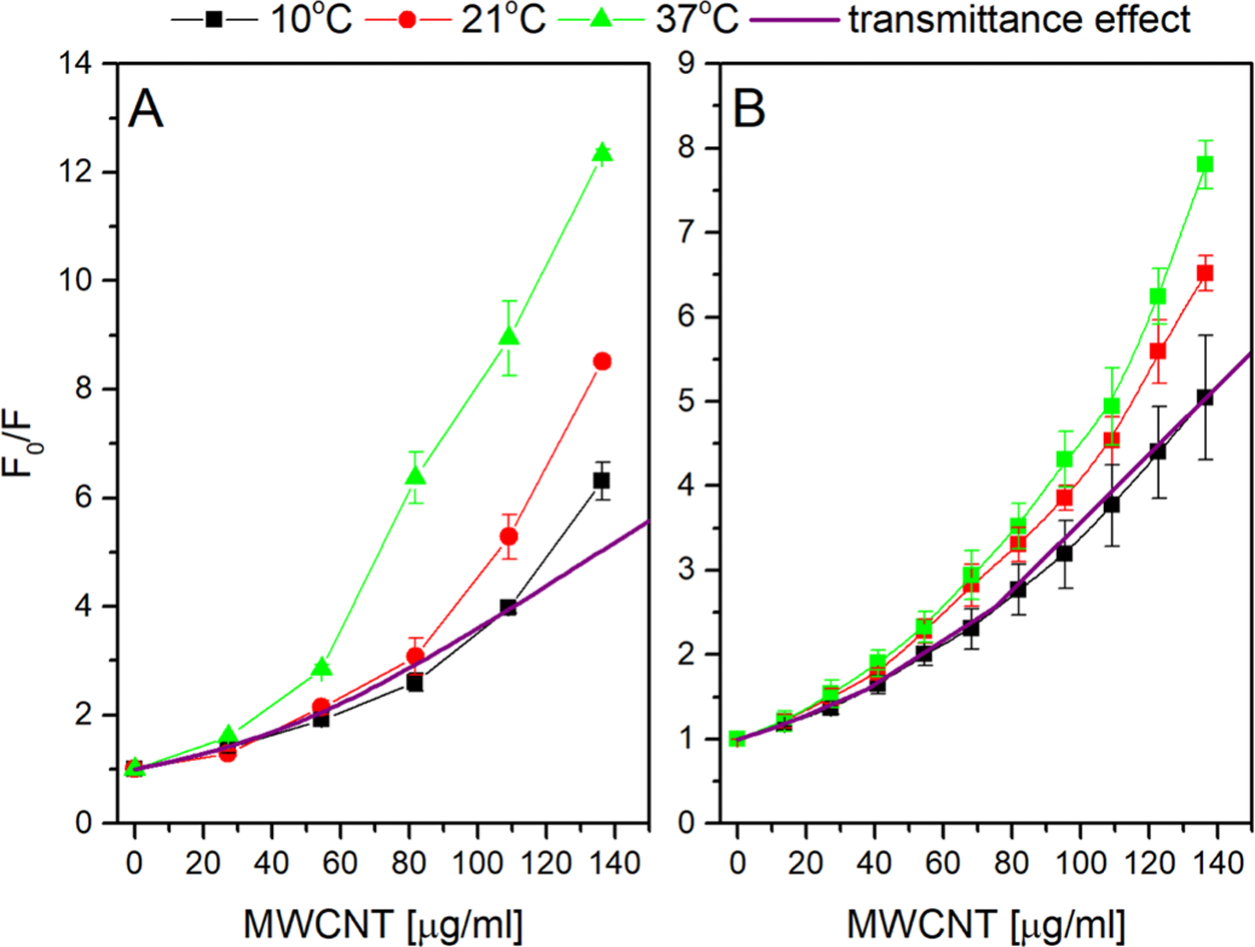
Quenching of PBSr (A) and LHCII (B) fluorescence by increasing the concentration of MWCNTs. For LHCII, excitation at 405 nm was followed by emission at 675 nm. For PBSr, emission was 650 nm with excitation at 575 nm. F0 is the fluorescence intensity without a quencher, and F—fluorescence intensities with increasing MWCNTs. Titration was done at ∼1 μM LHCII and 0.2 μM PBSr, respectively, in a final 1 mL of 25 mM HEPES/NaOH, pH 7.5, and MWCNTs were added in 1–2 μM aliquots from concentrated stock solution. The transmittance effect shows the hypothetical fluorescence intensity change if caused by MWCNT light screening only (see the text and Figure S1 for an explanation).

Here, CNTs are used as a platform for nanohybrid construction. It is known that CNTs may efficiently absorb light energy from various biological molecules attached to their surface. It results in, for example, the quenching of fluorophores. To establish the range of possibly unwanted interaction between photosynthetic antennae and CNTs without the covalent attachment, the changes in fluorescence emission of LHCII as a function of the MWCNT concentration were followed (Figure 1). A significant decrease in the fluorescence intensity was found. Figure 1A presents the Stern–Volmer relationship calculated for the collected data. Interestingly, the Stern–Volmer curve is not a straight line, which would indicate only one type of quenching. The curve reflects the weaker quenching in the lower concentrations of MWCNTs (in the range of 0–25 μg/mL of MWCNTs) and the stronger quenching in the higher concentrations of MWCNTs. PBSr titration exhibited similar behavior (Figure 1B), although the quenching was weaker. To conclude about the quenching mechanism, fluorescence lifetimes for LHCII/PBSr in the presence and absence of MWCNTs (Table 1) were compared. In the presence of MWCNTs, the τ values decreased significantly for final titration points (130 μg/mL MWCNT), while the change was measurable but weak for the 25 μg/mL MWCNT.

**Table 1:** Fluorescence Lifetimes [ns] Calculated for Particular Photosynthetic Antennae in the Presence of MWCNT (25 or 130 μg/mL)^a^

photosynthetic antennae type	no MWCNT	25 μg/mL MWCNT	130 μg/mL MWCNT
LHCII	0.60 ± 0.08	0.57 ± 0.02	0.52 ± 0.04
PBSr	1.72 ± 0.03	1.54 ± 0.16	0.72 ± 0.14
Zn-HP7	1.97 ± 0.18	1.13 ± 0.37	nd

a The errors represent standard deviations of at least three measurement repetitions. Results calculated from TCSPC decay curves, recorded for mixtures in a cuvette, thermostated at 22 °C.

As the temperature is a factor influencing quenching, the titrations were repeated at two additional temperature values (Figure 1). For PBSr, the extent of the quenching significantly increased with temperature, although the effect was apparent for only the higher MWCNT concentrations (Figure 1B). In the lower MWCNT concentration, the temperature effect was within the error range (not shown). The same was true for LHCII titration with the MWCNT (Figure 1B).

In the case of the artificial photosynthetic HP7 antenna protein, the titration was possible only for the lower MWCNT concentration, although a significant increase of quenching with temperature was noted (Figure S2). Additionally, the margins of error were large, especially at higher temperatures, indicating lower stability of the protein–pigment complex. For this reason, this protein was excluded from further preparations of nanohybrids.

As the transmission of MWCNT solution greatly decreased with the rising concentration, the simple absorption screen effect, caused by MWCNTs, was estimated from the change in transmittance in the function of nanotube concentration. The change was not linear in the whole tested concentration range (Figure S1) and led to a nonlinear Stern–Volmer relationship. The simulated “transmission effect” is also added to Figure 1. It is clear that for the lower MWCNT concentration, most (but not all, compared to the τ drop at 25 μg/mL, Table 1) of the observed quenching effects were caused by simple light screening, while for the higher MWCNT concentration, other mechanisms are in play. To minimize unwanted but possible energy transfer from QD or photosynthetic antennae to CNT, a BSA protein shell was therefore added to MWCNTs before further conjugation.

### Nanohybrid Composition Analysis

**Figure 2:**
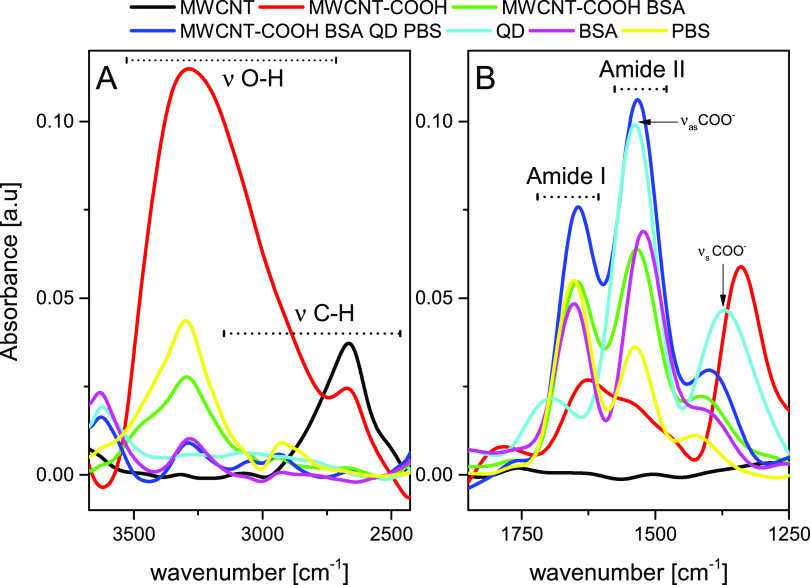
FT-IR analysis of the CNT conjugation process. (A) The OH and CH vibration range and (B) amide vibration range recorded for original MWCNTs, their oxidized version, as well as MWCNTs decorated with BSA and BSA-PBSr-QD530. BSA, PBSr, and QD530 spectra are shown for reference. No normalization was applied.

To evaluate the final nanohybrid construction, we tracked the following synthesis steps by FT-IR spectroscopy. Figure 2 presents representative examples of FT-IR spectra for MWCNTs and their oxidized versions, as well as MWCNTs decorated with BSA and BSA-PBSr-QD530. The same analysis for conjugation with LHCII is presented in Figure S3. The spectrum of the pristine CNT powder does not contain any strong bands, except vibration at about 2700 cm^–1^ (Figure 2A). This band might be interpreted as a C–H vibration, indicating the presence of some defects in CNTs. The oxidation procedure resulted in a broad band in the region 3000–3500 cm^–1^ (Figure 2A), which is attributed to the presence of the O–H vibrations, both hydroxyl and carboxyl ones. The attachment of proteins results in the narrowing of this band. The same narrow band is present in the pure BSA or PBSr spectrum, suggesting that original CNTs’ O–H vibrations are blocked following the conjugation. Characteristic fingerprints of proteins are present in the 1800–1250 cm^–1^ range (Figure 2B). The spectrum of original CNTs again does not show clear bands in this region. After oxidation, three bands appeared at 1760, 1600, and 1300 cm^–1^, which might be attributed to the COOH stretching vibrations. After the attachment of proteins, clear amide I (1650 cm^–1^) and amide II (1550 cm^–1^) bands were detected. The presence of QDs resulted in a change in the amide I/amide II intensity ratio as COOH asymmetric stretching (1550 cm^–1^) overlaps with the amide II band. In the case of a preparation containing the LHCII, the main difference is in the 275–3000 cm^–1^ range. This is the representation of lipids present in LHCII preparation. These bands are present in a final nanohybrid. One may note an increase in the intensity of the 2955 cm^–1^ band in comparison to the 2925 cm^–1^ band, representing a rise in detectable C–H stretching vibrations.

**Figure 3:**
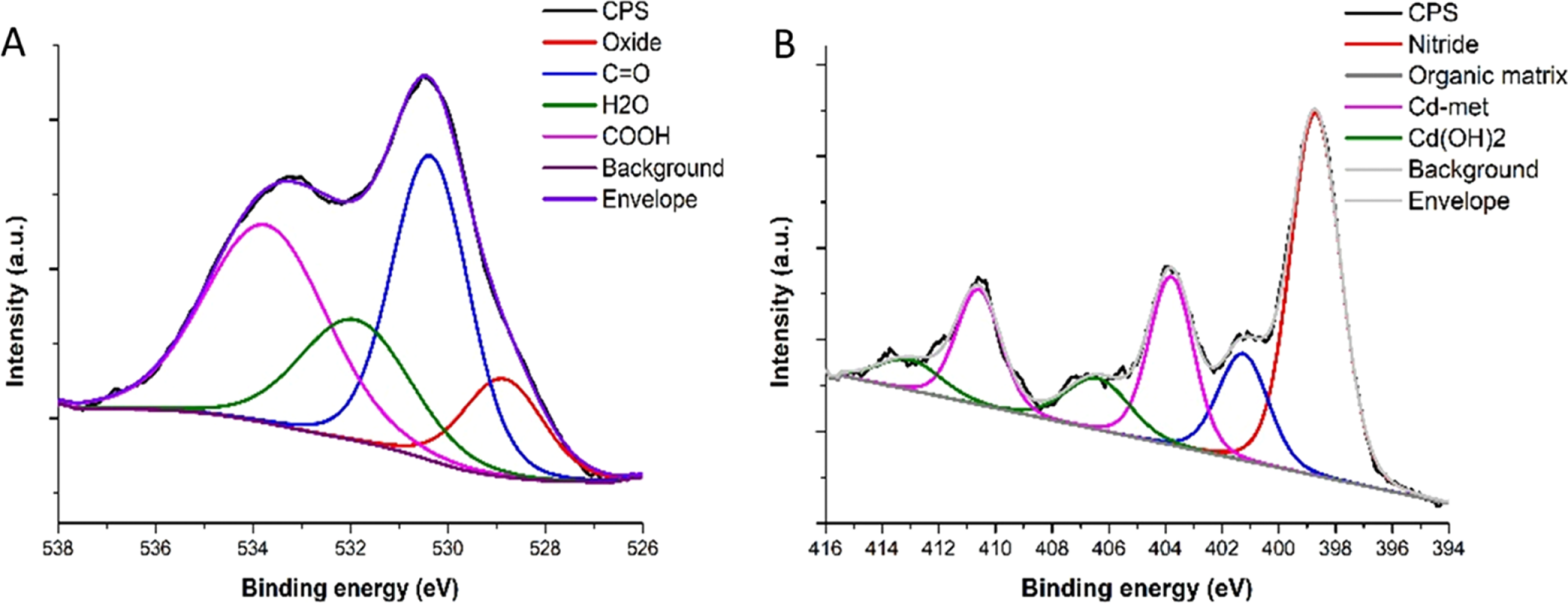
XPS analysis of MWCNT-BSA-QD preparation. (A) The region of O 1s with a clearly present COOH bond and (B) the region of N 1s with visible Cd bonds.

Clear confirmation of the QDs presence in the final nanohybrid, as well as the detailed elemental analysis, was possible thanks to XPS measurement (Figure 3 and Table S1). First, the presence of COOH in the samples after conjugation of CNTs with BSA and later subsequently with QDs was confirmed by the presence of COOH bonds in the O 1s and C 1s regions presented as an example in Figure 3A. The confirmation of the conjunction of BSA and QD can be observed in the N 1s region, where the overlapping of the Cd 3d region appears in Figure 3B. In the QD-containing sample, clear bands at ∼404 and ∼411 eV were detected, with Δ*E* = 6.79 eV, which is characteristic of CdTe. Also, in the Te 3d region, the presence of a CdTe bond at around 573 eV was detected, not shown in the present paper. Also, QD addition resulted in the detection of sulfur coming from the β-mercaptopropionic acid coating of QDs. Sulfur is also present in BSA, but its content might be too low for detection. Surprisingly, Fe, Ni, Na, and Co were detected in the preparation of oxidized CNTs, while in a pristine CNT preparation, there were only carbon and oxygen. These atoms may come from impurities in piranha and NaOH used for neutralization. After conjugation with BSA, all of these elements (except Fe) were absent, indicating that the metal cations were bound to the ionized COOH groups. After EDC/NHS treatment, no more COOH groups were available for the coordination of additional ions. Interestingly, the attachment of BSA resulted in the detection of Cu and did not eliminate the Fe. The BSA protein is known for copper^37,38^ and iron^39^ coordination, which explains their detection.

### Energy Transfer Analysis: Confirmation of the Interaction between Elements Immobilized on a Carbon Nanotube Platform

To verify that QDs and photosynthetic antennae may interact with each other and, therefore, the constructed nanobiohybrid has the desired properties, we focus on the observation of resonance energy transfer.

For FRET characteristics of the studied donor–acceptor pairs, see Table S1 and Figure S4. As fluorescence signals were too weak for measurement in a cuvette (due to the screening effects of CNTs described earlier), CLSM analysis was done, allowing for the detection of a signal from a single layer of nanohybrids deposited on a cover glass. Figures S5 and 6 present examples of CLSM and FLIM images, recorded for various preparations of nanohybrids with PBSr, obtained during this research. The example for MWCNT-BSA-QD570-LHCII preparation CLSM images is shown in Figure S7. In all cases, both QD and LHCII/PBSr can be detected on an MWCNT surface. Fluorescence spectra, extracted from CLSM data, are in agreement with the expected fluorescence emission maxima of respective fluorophores (Figure S8). In an analysis of this type, however, it is impossible to deduce actual change in the fluorescence intensity; therefore, the conclusions were based on a FLIM analysis.

The calculated fluorescence lifetime values (Tables 2 and 3) show the increase of, respectively, the LHCII lifetime in the MWCNT-QD570-LHCII and the PBSr lifetime in the MWCNT-QD530-PBSr nanohybrid in comparison to the MWCNT-LHCII/PBSr nanohybrid. This is the expected indication of the energy transfer from QD570 to LHCII and from QD530 to PBSr. The QD570 lifetime in the MWCNT-QD530-LHCII nanohybrid was, contrarily to the expectation, longer than in the MWCNT-QD570 nanohybrid. This effect might be explained when the MWCNT-QD570 lifetime is compared to the lifetime of QD530 in solution; the value is shortened, which suggests some aggregation of QDs or the still possible energy transfer to a CNT. The presence of LHCII may prevent such an aggregation; therefore, the measured τ value of QD570 in MWCNT-QD570-LHCII was still higher than in MWCNT-QD570.

**Table 2:** Fluorescence Lifetimes (ns) Calculated for Particular Components of the CNT-QD570-LHCII Nanohybrid Based on FLIM^a^

	emission range	
	520–587 nm	702–799 nm
LHCII		0.29 ± 0.01
CNT-LHCII		0.25 ± 0.02
CNT-QD570-LHCII	4.312 ± 0.72	0.99 ± 0.19
CNT-QD570	2.85 ± 0.64	
QD570	29.46 ± 0.26	

a Possible tail emissions of QDs in 702–799 nm were measured and subtracted. The error represents the standard deviation of at least 15 separate ROIs.

**Table 3:** Fluorescence Lifetimes [Nanoseconds] were Calculated for Particular MWCNT-QD530-PBSr Components and Chlorophyll, alone or in a Mixture, Respectively, in the Presence or Absence of Thylakoids^a^

	emission range		
	510–549 nm	625–667 nm	700–799 nm
*PBSr		1.68 ± 0.02	
MWCNT-PBSr		0.82 ± 0.07	
*QD530	25.67 ± 0.36		
MWCNT-QD530	3.08 ± 0.79		
MWCNT-QD530-PBSr	2.65 ± 0.62	1.48 ± 0.45	
thylakoids			0.45 ± 0.09 (ex 665 nm)
			0.23 ± 0.07 (ex 500 nm)
MWCNT-QD530-PBSr + thylakoids	0.65 ± 0.22	0.68 ± 0.10	0.64 ± 0.27 (ex 500 nm)
MWCNT-QD530 + thylakoids	2.65 ± 0.42		0.45 ± 0.01 (ex 500 nm)

a Lifetimes of the components of MWCNT-QD530-PBS and MWCNT-QD530-PBS+thylakoids samples were calculated from the selected ROIs that were overlapped in 80–100% of the surface with ROIs from the other emission channels. Possible tail emissions of QDs in 625–667 nm and 700–799 nm were measured and subtracted. The error represents the standard deviation of at least 15 separate ROIs. The lifetimes for PBSr and QD530 alone (triplicates, 1 μM concentrations, marked by *) were measured in the droplet of solution as a homogenous FLIM image (ROIs were not extracted).

FLIM imaging was also used to demonstrate the actual energy transfer from CNT-bound QD530 via PBSr to chlorophyll molecules present in photosynthetic complexes in thylakoids isolated from *Synechocystis* PCC 6803. The thylakoids were mostly depleted from PBSr; therefore, CNT-bound PBSr might be in a position to transfer its excitation to reaction centers. CLSM images of the MWCNT-QD530-PBSr mixture with thylakoids revealed that the thylakoids spontaneously assembled around CNTs. This might be due to multiple types of interaction, including electrostatic and hydrophobic bonding. In this system, further shortening of QD530 and PBSr τ values was observed while the lifetime corresponding to chlorophyll emission was increased. Without PBSr, the chance of nanohybrid:thylakoid binding was greatly decreased (see Figure S9), and there was no increase in a chlorophyll fluorescence lifetime.

**Figure 4:**
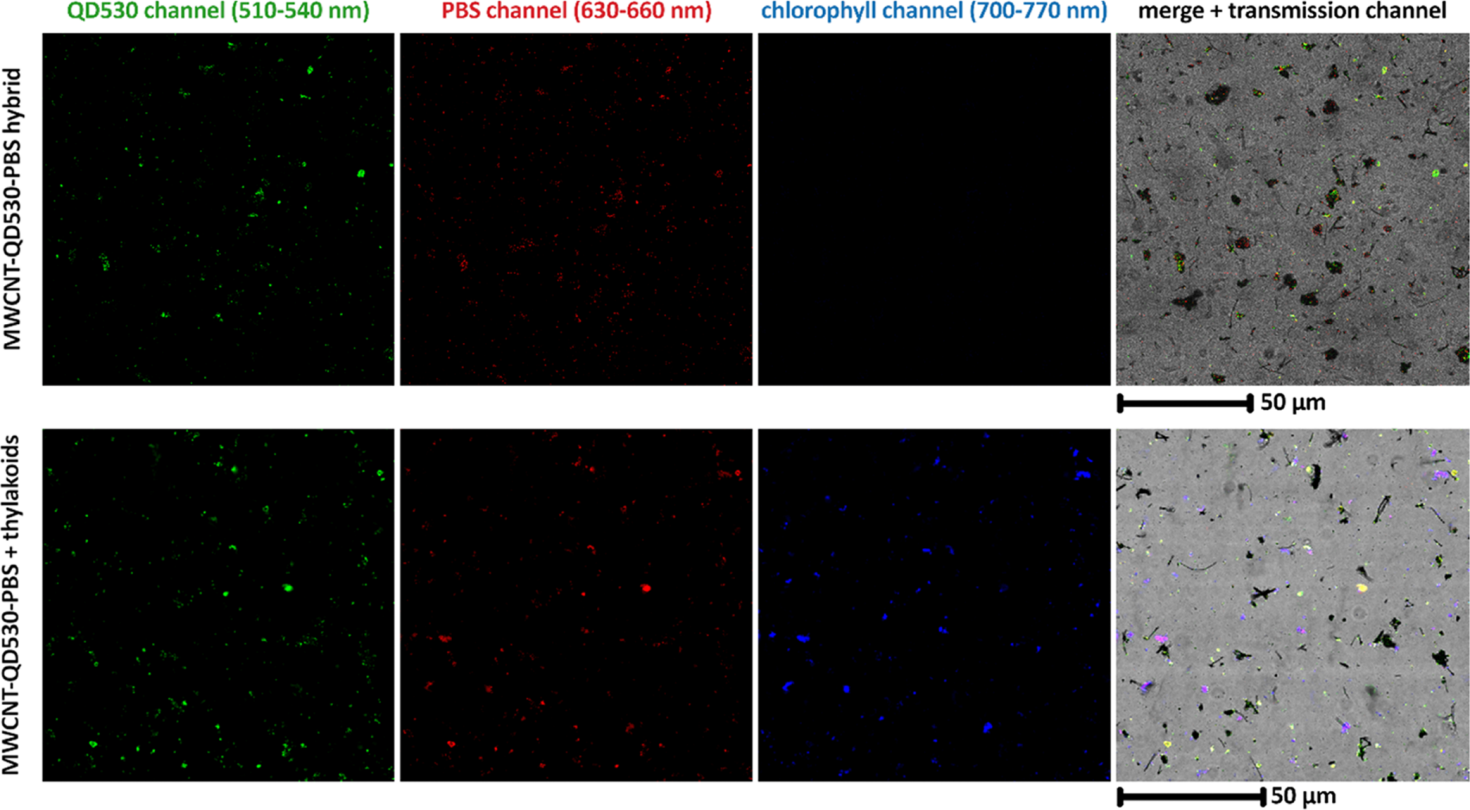
CLSM images of MWCNT-QD530-PBSr hybrids and their mixture with *Synechocystis* PCC 6803 thylakoids. The samples contained MWCNT-QD530-PBSr (∼300 μg/mL) with or without thylakoids (the amount corresponding to 100 μg/mL of chlorophyll). The excitation wavelength was 500 nm, and the emission ranges for each channel are indicated.

**Figure 5:**
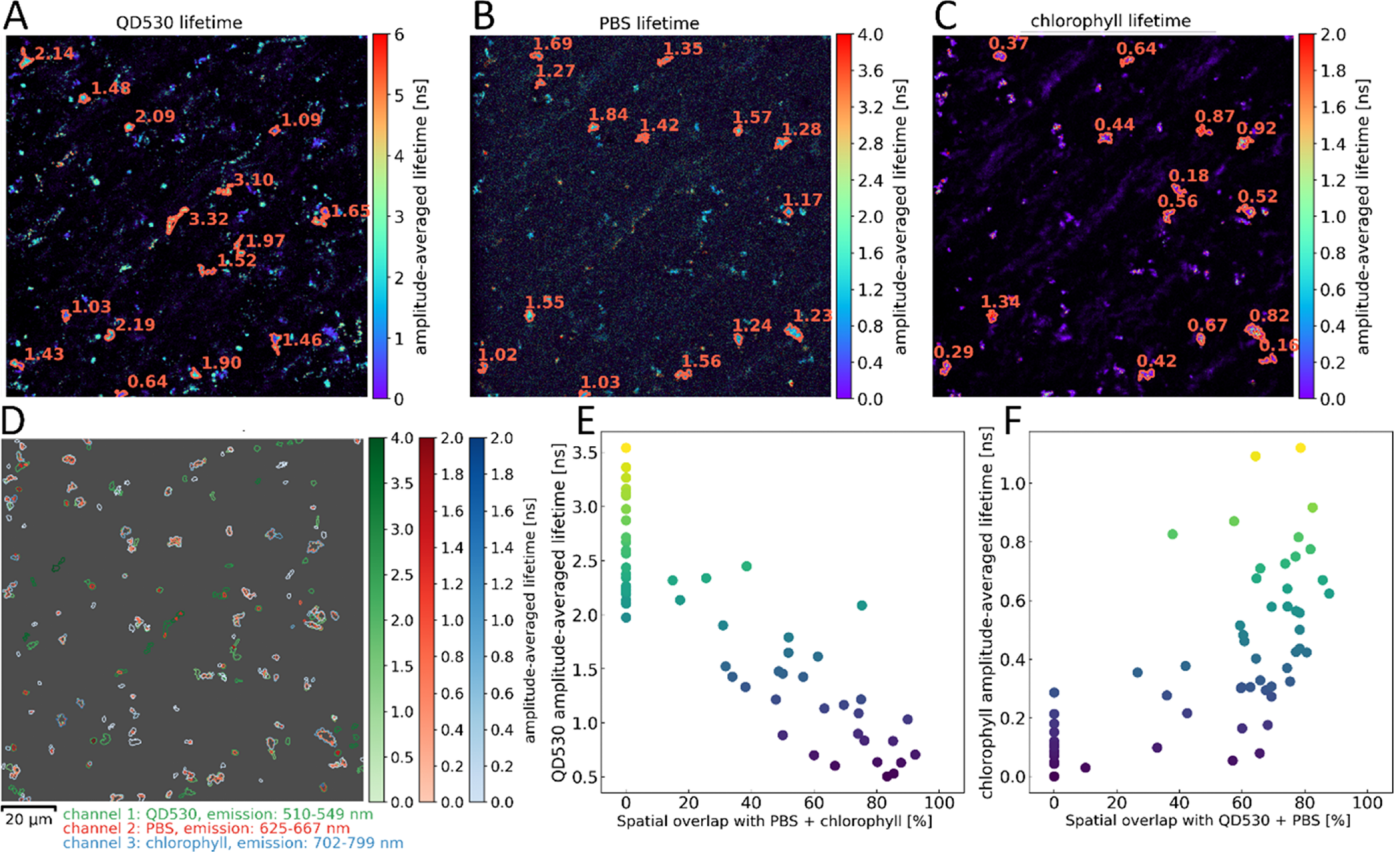
Representative example of the FLIM analysis for MWCNT-QD530-PBSr (∼300 μg/mL) and thylakoid mixtures (100 μg/mL of chlorophyll). (A–C) FLIM images of QD530, PBSr, and thylakoid, respectively. Averaged lifetimes [ns] for selected ROIs are indicated. The excitation wavelength was 500 nm, and the emission ranges for each channel were listed. (D) The overlap image of ROIs extracted from FLIM images. The contours of the individual ROIs are colored according to their lifetime. (E, F) The dependence of QD530 and the chlorophyll lifetime on the spatial overlap with the other components of the FRET system. The points represent individual ROIs, colored according to the lifetime. For the details of the analysis procedure, see the Supporting Information, Figures S5 and S6.

The analysis of the spatial overlap of ROIs corresponding to three separate channels of FLIM images (QD530, PBSr, and chlorophyll of thylakoid membranes) showed the significant dependence of an emission lifetime on the colocalization of the elements of the MWCNT-QD530-PBSr system. In the presence of thylakoids, the QD530 lifetime decreases when their emission overlaps with the emission of thylakoids, and reversely, the lifetime of chlorophyll increases when the thylakoids are in close proximity to MWCNT-QD530-PBSr. The lifetime of PBSr in this system is longer or shorter depending on the respective role of PBSr in the system—an FRET acceptor (when colocalized with QD530) or donor (when colocalized with thylakoids) (Figures S5 and S6). The lifetime of PBSr is constant in the ROIs that colocalize with both QD530 and thylakoids when PBSr functions as an intermediate transmitter of resonance energy between QD530 and chlorophyll. Overall, the analysis of FLIM images confirms the sequential FRET in our system in the direction QD530 → PBSr → chlorophyll (Figures 4 and 5).

### High-Resolution Nanohybrid Imaging: Additional Checkup of a Nanobiohybrid Construction

**Figure 6:**
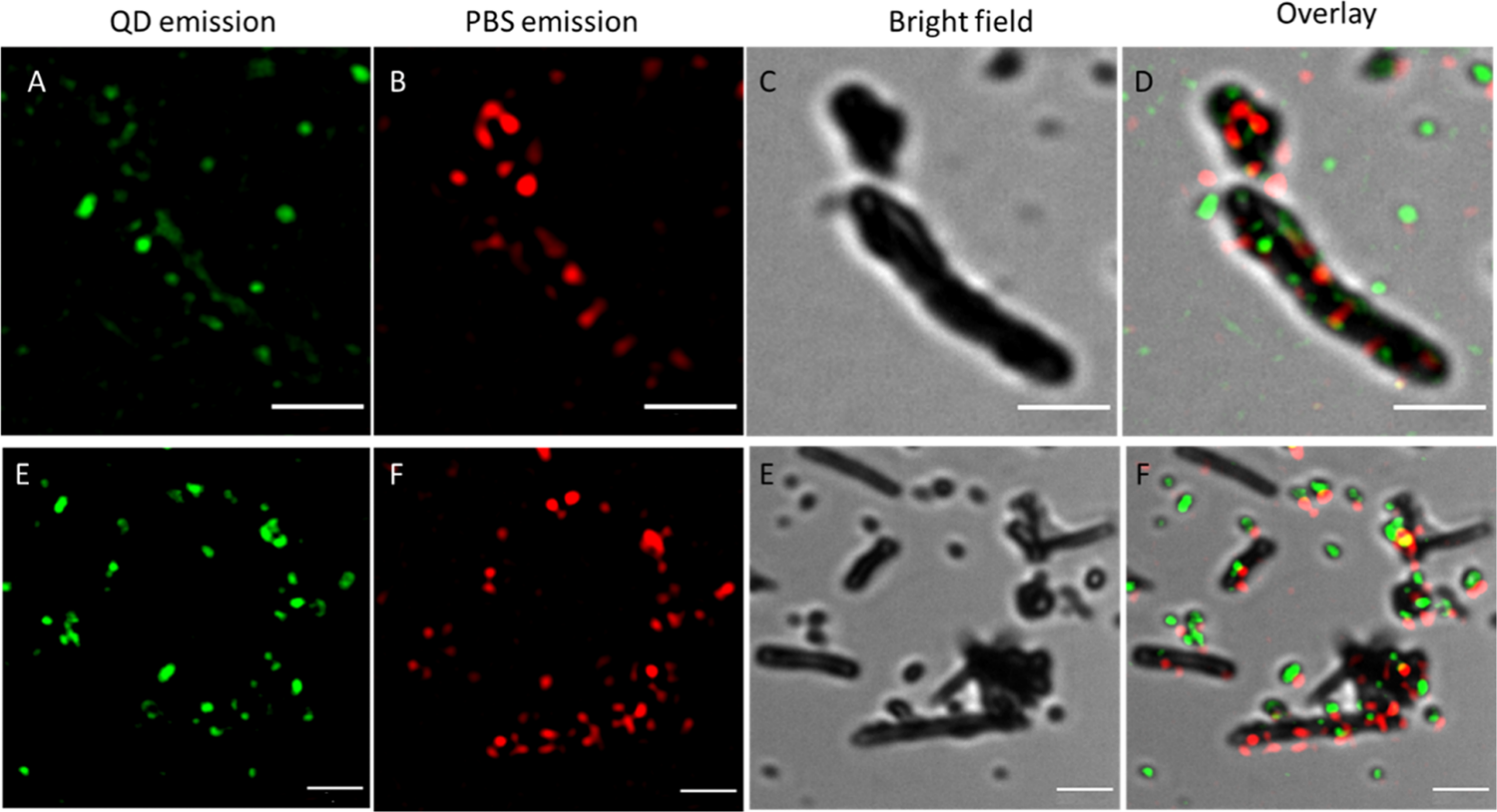
Green channel (A, E)—SIM_2_ image of QD530 fluorescence (excitation 405 nm, emission 490–570 BP), red channel (B, F)—SIM_2_ image of PBSr fluorescence (excitation 630 nm, emission with FS filter), transmission channel (C, G) normal resolution, no processing) and overlay (D, G) of channels. Scale bar = 2 μm.

For further verification of obtained nanobiohybrids, their shape and surface characteristics were evaluated by high-resolution imaging with atomic force microscopy (AFM) and SIM^2^ CLSM technique. Using AFM, we were able to show that the surface of SWCNTs had a lot of bulges after the full decoration procedure (Figure S10). SWCNTS were used here instead of MWCNTs, as the chemistry of their surface is identical, and optimization of AFM imaging resulted in clearer results. The bulges were absent in BSA-coated SWCNTs; therefore, they might be attributed to PBSr, as the size of this complex is much bigger than that of a BSA molecule or a single QD nanoparticle. Although we may conclude about the identity of the SWCNT-attached particles from their size, the AFM data does not allow for a simple differentiation between PBSr and QDs. To learn more about PBSr and QD distribution on the nanotubes, the SIM^2^ CLSM technique, with a fluorescence image of a resolution estimated at 60 nm, was applied. Figure 6 presents examples of images recorded for fully decorated MWCNTs. Both QD530-related and PBSr-related emission pixels were clearly located on an MWCNT. Significantly, in multiple places, both signals colocalized, indicating QD530 and PBSr attachment in close proximity to each other. It might also be noted that shorter MWCNTs seem to be decorated with a higher yield. This is probably a result of the higher number of defects of the graphene lattice on the MWCNT edges, which might be easier to oxidize and introduce COOH groups, being the points of the following attachment.

### Obtained Nanobiohybrids may be Included in Longer Energy Transfer Chains: Energy Transfer to the Photosynthetic Reaction Center Assessed by Methyl Viologen Reduction

**Figure 7:**
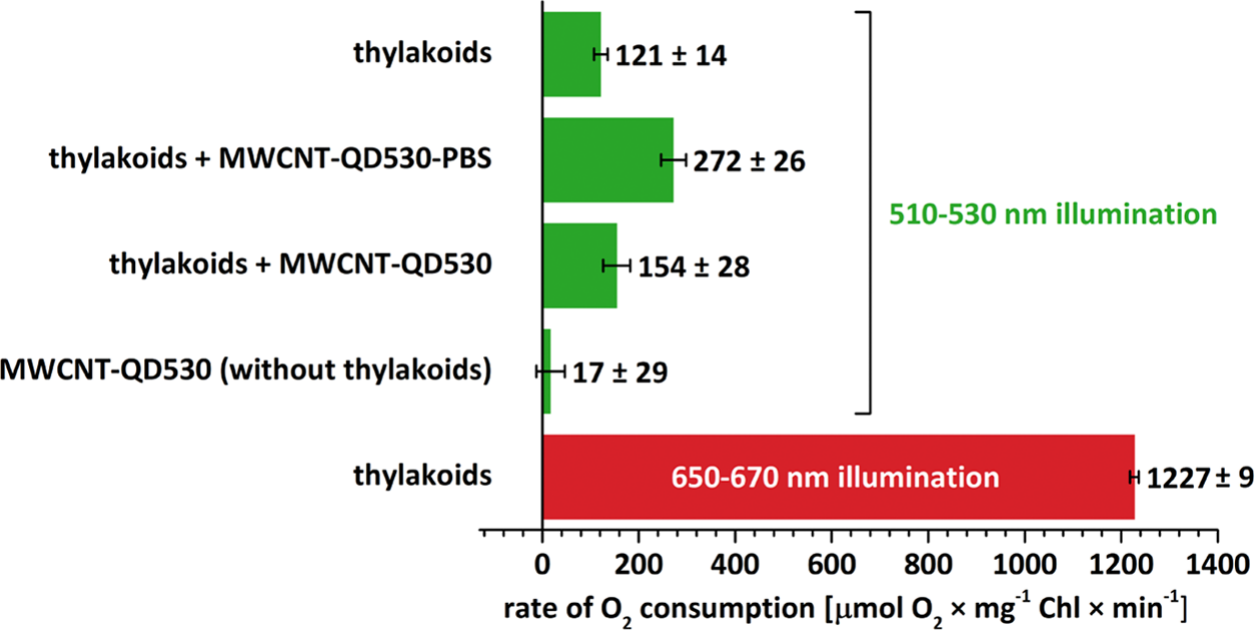
Light-dependent oxygen consumption by thylakoids is greatly enhanced in the presence of the MWCNT-BSA-QD530-PBSr nanohybrid. The samples contained the number of thylakoids corresponding to 20 μg/mL chlorophyll and ∼400 μg/mL nanohybrid in the presence of a redox system (1 mM sodium ascorbate +0.1 mM DCPIP + 0.5 mM methyl viologen). The rate of oxygen consumption was calculated according to the slopes of oxygen concentration curves measured by the Clark-type electrode during the first 2–3 min after turning on the illumination.

To prove that components joined on a carbon nanotube platform might be included in longer energy transfer chains, thylakoids with native photosynthetic machinery were used as final acceptors. The energy transfer from QD530 to thylakoids, via the CNT-attached PBSr, was probed by the observation of oxygen consumption enhancement. Figure 7 presents the comparison of oxygen consumption rates measured for various mixture compositions, while Figure S11 shows examples of recorded traces. When thylakoids alone were illuminated using a 510–530 nm band-pass filter (chlorophyll absorption minimum), the reaction was much slower than for thylakoids illuminated in the range 650–670 nm (chlorophyll absorption maximum). However, in the presence of MWCNT-QD530-PBSr, the oxygen consumption for the 510–530 nm illumination range increased almost twice. The effect was caused by the interaction between a nanohybrid and thylakoids, as MWCNT-QD530-PBSr alone did not influence the oxygen level directly or by a photoinduced MV reduction.

## Discussion

The nanohybrid materials have great potential as nanobiodevices, components of alternative energy sources, and catalysts.^3,29,30,40−44^ Carbon nanotubes, as a base for such a construction, may be a neutral nanosized platform or an active component working as the final energy sink or the electron acceptor. Here, we exploited the first option by demonstrating the junction of QDs and photosynthetic antennae on the surface of a CNT, further proving that energy from an excited QD may form a functional connection, further able to power photosynthetic reactions in a thylakoid via QD-PBSr-thylakoid energy transfer. Photosynthetic proteins of various origins are often considered as parts of green energy devices or other nanohybrids.^7,29^ Usually, those are just two-component hybrids, such as PSI and carbon nanotube only;^26−30^ however, multicomponent junctions were also explored^8,45^

Multiple research suggests that carbon nanomaterials may efficiently steal fluorescence energy from the excited molecules.^31,46−48^ To establish the probability and efficiency of this process in our system, the quenching of the fluorescence emission of the photosynthetic antennae upon titration with the increasing concentration of CNTs was estimated. The fluorescence intensities of LHCII, PBSr, and the model photosynthetic antenna decreased with the increase in the quencher concentration. However, up to about 25 μg/mL CNTs, the effect was mostly related to a light screening, the drop of the light intensity reaching photosynthetic antennae due to its absorption by CNTs. More prompt effects were observed for higher amounts of CNTs. In the case of protein maquettes, the effects were strongest and indicated the instability of the ligand–protein complex in the presence of CNTs. It might be due to a relatively small protein size and many of the possible interaction points on a CNT surface. LHCII and PBSr, being vast and more stable complexes, seemed to be more promising candidates for a further junction.

Due to the possibility of excitation energy stealing by MWCNTs in a broad range of concentrations, even without covalent binding of the antenna complexes, a shell distancing the CNT surface from the other nanohybrid components was added. BSA was chosen for that purpose, being a protein known to be neutral and relatively stable in many biological applications, especially in surface passivation.^49^ BSA shape might be approximated by an ellipsoid with an effective diameter of about 7 nm.^50^ With a single layer of BSA molecules, the distance is not large enough to completely prevent FRET from reaching MWCNT, but its probability greatly decreases. The addition of BSA as a first layer may also statistically increase the success of the following attachment steps, as the protein molecule has more than one accessible carboxyl and amino group. Additionally, BSA acts as a linker between a QD and a CNT, as both CNTs and QDs have carboxyl-covered surfaces, and there is no simple chemistry available for the carboxyl-carboxyl bond formation.

Using the procedure elaborated here, QDs and PBSr/LHCII were conjugated on an MWCNT surface in a manner that allowed them to form an energy transfer pair. For both photosynthetic antennae, QD with optimal emission properties was used (QD530 or QD570 for PBSr and LHCII, respectively), prompting efficient energy transfer if only a close distance between the donor and the acceptor was assured. QDs were already shown to be able to donate energy to PBSr located in a short enough proximity.^32,33^ The energy transfer in our system was proven by FLIM measurement, showing the fingerprint of FRET, the increase in acceptor τ. This technique was successfully used for proving protein–protein^51^ as well as protein–nanoparticle interaction.^33^

The covalent binding fixes the components of the nanohybrid assembly in place. However, the distribution of components is stochastic, which may result in spatial gaps preventing resonance energy transfer. To be sure that the distances between components will be short enough, excess reagents during chemical conjugation are necessary. Judging from confocal microscopy images, the surfaces of shorter nanotubes seem to be covered almost completely by the fluorescence signal. One needs to remember, however, that a recorded fluorescence area is usually higher than an area of fluorophore, and the coverage may not be ideal. The longer CNTs tend to have quite a lot of empty spaces (compare Figure 6). This might be due to the lower effectiveness of oxidation and, as a consequence, a lower number of available carboxyl groups. What is important, even with nonfull coverage, QDs and PBSr antennae fluorescence signals colocalize in multiple places, indicating efficient binding of both the donor and the acceptor in the same spot on the CNT surface.

Finally, the ability of the CNT-QD-PBSr nanohybrid to power natural photosynthetic systems was tested. As an example, photosynthetic membranes (thylakoids) isolated from cyanobacterium *Synechocystis* PCC 6803 were chosen. During their isolation procedures, the membranes form small vesicles with active photosystems I and II (PSI and PSII). Based on the similarities to higher plants, the isolated cyanobacterial thylakoids presumably have a diameter of a few hundred nanometers.^52^ The isolated membranes are, however, mostly depleted of natural PBS antennae. Therefore, this is an opening for interaction with external PBSr. Such binding is possible since PBS is known to diffuse on the thylakoid membrane surface.^53^

The nanohybrid containing PBSr was chosen for these tests for two reasons. First, since both LHCII and photosystems contain chlorophyll molecules, it is virtually impossible to discriminate their emission at room temperature. Therefore, it is simply impossible to show that energy is transferred via LHCII and not from the QD to PSI/PSII directly. Second, PSI/PSII binding places for PBSr are available from the external aqueous environment, contrary to LHCII available from the membrane interior. It makes the interaction between CNT-bound PBSr and PSI/PSII of thylakoids more likely. For the interaction of LHCII with the photosystem, the nanohybrid would have to penetrate the membrane. It is not impossible (see research on thylakoids covering CNT^23,25^), but simpler interaction gives more chances for success.

In the presence of thylakoids, after excitation by a properly selected light wavelength, CNT-bound QD expects to serve as a donor, PBSr as a mediator, and a final acceptor is a chlorophyll within photosystems. This assumption corresponded to changes in the fluorescence lifetimes of the system components observed in FLIM experiments: QD and PBSr τ values were shortened, while chlorophyll τ was increased. Through the oxygen electrode experiments, we tested PSI activity. It was done indirectly by observing methyl viologen (MV) reoxidation. MV is reduced by PSI in a light-dependent manner with ascorbate and DCPIP as a sacrificial reductant system. Without light, the system is inactive. In this study, when green light was used as the illumination source, the PSI activity was minimal. The situation changed when nanohybrids were added; QDs, absorbing green light, donated energy downhill through the nanohybrid-PSI chain to power the MV reduction.

**Figure 8:**
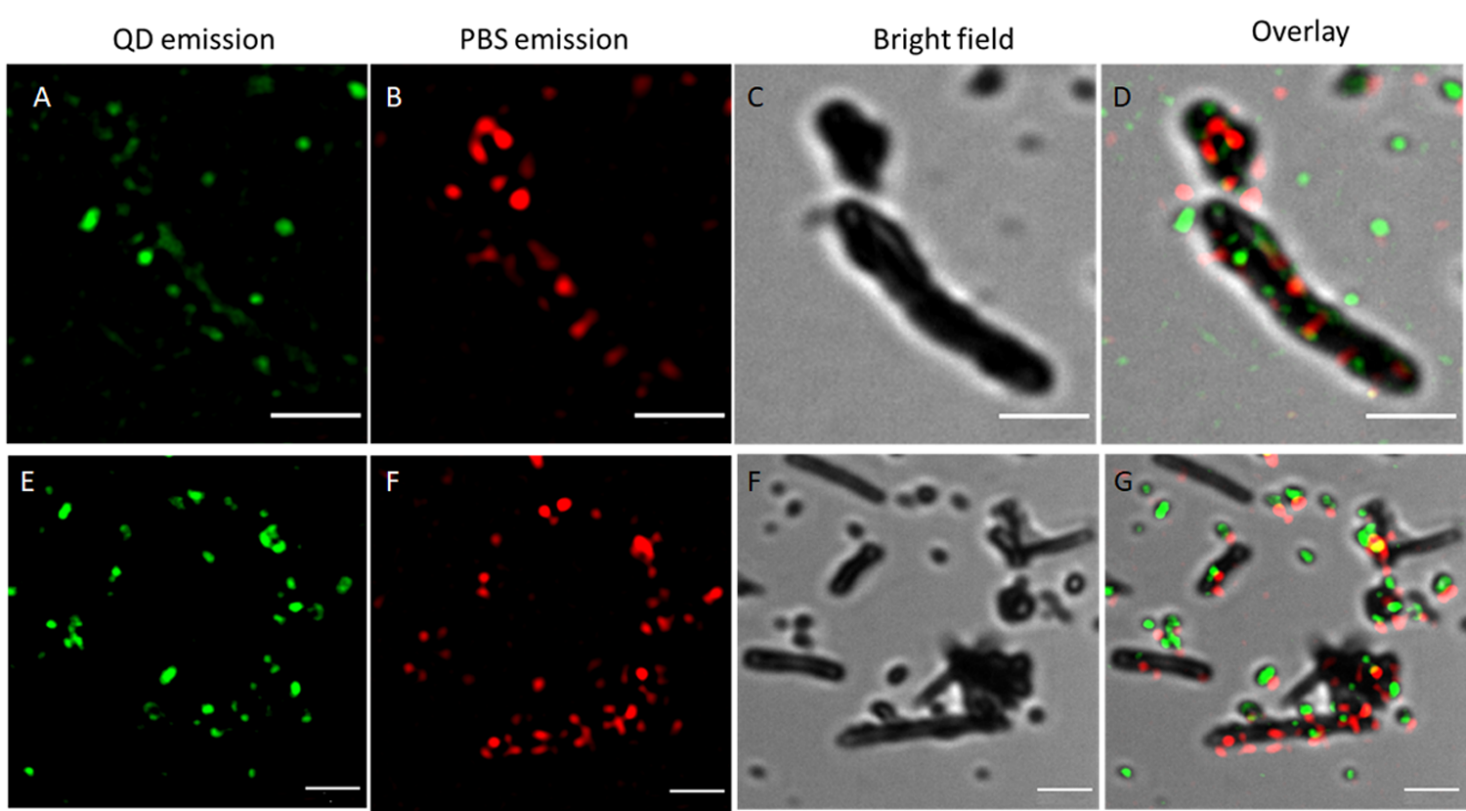
Schematic representation of the possible routes of energy transfer in a system composed of CNT-QD-PBSr and thylakoids (A) and differentiation of the thylakoid photosystems according to the spatial distance to a nanohybrid and access to energy transfer donors. Note that the scheme does not preserve the size ratio of combined elements and may not represent exactly the actual arrangement of elements.

Considering the effectiveness of the energy transfer to photosystems, one should remember that the colocalization between QD-PBSr and PSI in photosynthetic membranes is stochastic. However, when PSI complexes are densely packed in photosynthetic membranes (with a mutual distance smaller than 50 nm^54^), the probability of functional connection increases. Due to certain diffusional freedom of pigment–protein complexes within thylakoid membranes,^55^ their partial spatial adaptation to the PBSr position on CNT is possible. Nevertheless, under our experimental conditions, a huge surface of thylakoids is devoid of contact with CNTs and, therefore, without a chance of energy donation from QD (Figure 8A,B). Additionally, it needs to be remembered that PBSr cannot transfer energy to PSI with that effectivity, as would be achieved with allophycocyanin (a connector) present in the system. As we are observing more efficient nanohybrid–thylakoid interaction when PBSr is present as part of a nanohybrid, some allophycocyanin may be left on thylakoids, therefore participating in mediating the energy transfer. However, this again is with a certain dose of probability and may result in the final observed effectiveness of measured processes. It also needs to be remembered that PBS might be connected both with PSI^56^ and PSII,^57^ while there is a great excess of PSI in cyanobacterial membranes (with a ratio reaching 5:1).^58^ This may increase the chance of nanohybrid binding with PSI. Additionally, most of the chlorophyll fluorescence of cyanobacterial membrane would result from PSII emission.^59^ Therefore, it may be easier to note the increase of oxygen consumption, being here the way to assay the PSI activity, than to observe a relatively small increase in the chlorophyll fluorescence, which would result from the energy transfer to PSII.

The system we showed here is an example of a nanohybrid that enables the enhancement of light energy absorption and is the preliminary basis for multiple possibilities and improvements. First, it allows for the diversity of final acceptors powering different chemical and enzymatic reactions. The donor–acceptor relationship might be reversed with QDs working as the energy acceptor. Furthermore, one may take the opportunity to use the QD-powered nanohybrid system not only regarding the FRET process but also employing the photoinduced electron transfer from QD. Finally, using CNTs as scaffolds and assembly platforms, it is possible to exploit their electrical properties (e.g., high conductance) to follow or modify the nanohybrid-dependent reactions.

## Supporting Information Available

Details of FLIM analysis with representative images; FRET characteristics of studied nanobiohybrids and their components; atomic concentration of studied nanohybrids; transmittance changes in the function of CNT concentration; examples of absorption spectra of studied antennae pigment–protein complexes; FT-IR analysis of LHCII–carbon nanotubes complex; CLSM images of carbon nanotubes–LHCII complex in connection with thylakoids; examples of AFM images of carbon nanotubes–BSA–PSBr nanobiohybrid; and example traces illustrating thylakoid photosynthetic activity in the presence and absence of nanobiohybrids (PDF)
