# Correction to “Quantum Dots Assembled with Photosynthetic Antennae on a Carbon Nanotube Platform: A Nanohybrid for the Enhancement of Light Energy Harvesting”

**DOI:** 10.1021/acsomega.3c09478

**Published:** 2024-01-05

**Authors:** Jakub Sławski, Jan Maciejewski, Rafał Szukiewicz, Katarzyna Gieczewska, Joanna Grzyb

Description of correction being made to the manuscript file:

Update labeling of Figure 6: it was A, B, C, D, E, F, F, G now it is A, B, C, D, E, F, G, H

**Figure 6:**
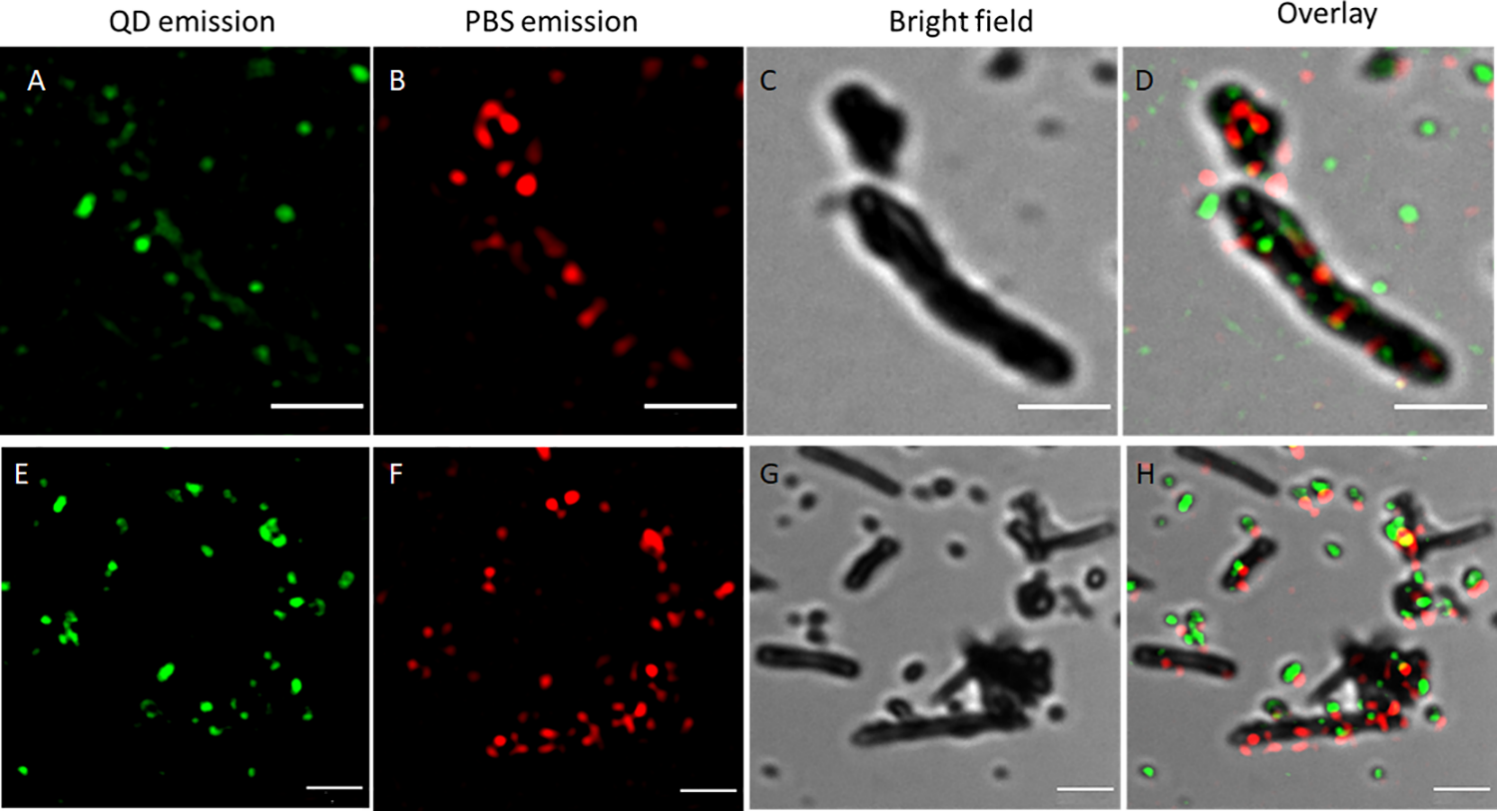


Corrected Figure 6

