# Supplementary material for: Quantum Dots Assembled with Photosynthetic Antennae on a Carbon Nanotube Platform: A Nanohybrid for the Enhancement of Light Energy Harvesting
Source: ACS Omega. 2023 Oct 26;8(44):41991–2003. doi: 10.1021/acsomega.3c07673 (PMC10633852; doi:10.1021/acsomega.3c07673)
Supplement: Supplementary file 1 — ao3c07673_si_001.pdf [file ao3c07673_si_001.pdf]

Supplementary materials

Quantum dots assembled with photosynthetic antennae on a carbon nanotube platform - a nanohybrid for enhancement of light energy harvesting

Jakub Sławski[1], Jan Maciejewski[1], Rafał Szukiewicz[2], Katarzyna Gieczewska[3], Joanna Grzyb[1]*

[1]Department of Biophysics, Faculty of Biotechnology, University of Wrocław, F. Joliot-Curie 14a, 50-383 Wrocław, Poland

[2]Faculty of Physics and Astronomy, University of Wrocław, Maxa Borna 9, 50-204 Wrocław, Poland

[3]Department of Plant Anatomy and Cytology, Institute of Experimental Plant Biology and Biotechnology, Faculty of Biology, University of Warsaw, I. Miecznikowa 1, 02-096 Warsaw, Poland

*corresponding author: joanna.grzyb@uwr.edu.pl

Analysis of FLIM images

The preliminary analysis of FLIM images was performed using LasX software (Leica, Germany) of the confocal system. The tail of the distribution curve of photon counts collected from the overall image was fitted with a four-exponential decay equation:

$$I(t) = I_0 + \sum_{i=1}^{4} A_i \times \exp\left(-\frac{t-t_0}{\tau_i}\right)$$

where $I(t)$ is the number of photon counts in the time t, I_0 is the tail offset (background), A_i is the amplitude of the i-th component associated with the τ_i lifetime, and t_0 is the time offset. Then, the lifetime values obtained from the fit were used to fit the images – the photon distribution for each pixel was fitted to the equation above with the lifetime values fixed. As the result, four amplitude images, each associated with a different lifetime component, were calculated.

The next steps of the analysis were performed using a self-written Python script. Four calculated lifetimes and the amplitude values corresponding to each image pixel were used to calculate the amplitude-weighted mean lifetime (τ_{av}) image, according to the equation:

$$\tau_{av} = \frac{\sum_{i=1}^{4} A_i \tau_i}{\sum_{i=1}^{4} A_i}$$

The set of two-pixel matrices – the emission intensity image (recorded by the confocal system) and the calculated mean lifetime image – were used to extract the ROI areas. Using the threshold corresponding to 50-70% of the maximum intensity value (the brightest pixel of the intensity image; the threshold was set depending on the background noise level), the mask was created to remove the pixels corresponding to the low-intensity background. The remained image consisted of irregular patches which were detected by the contour-finding algorithm (imported from the scikit-image library [1]) and sorted according to the surface area (in pixels). 50-100 patches of the largest area were selected as individual ROIs in each FLIM image.

The spatial overlaps for each ROI were calculated as the fraction:

$$\text{overlap} = \frac{\text{number of ROI pixels overlapping with ROIs from the other channel}}{\text{ROI area [pixels]}}$$

The overlap with the pairs of the channels (e.g. QD530+PBS) was calculated using the pixels shared by both channels (the intersection of both sets of pixels).

Table S1. FRET characteristics (overlap integral, J, and Förster radius, R_0) for the studied nanohybrids calculated based on original absorption and emission spectra obtained during this study. Calculation made in a|e- UV-Vis Spectral Software (http://www.fluortools.com).

Donor-acceptor pair	J(λ) [$nm^4M^{-1}cm^{-1}$]	R_0 [nm]
QD530-PBS	2.21×10^{16}	7.89
QD570-LHCII	3.31×10^{16}	7.56

Table S2. Atomic concentrations [%], estimated by XPS, in CNT and its nanohybrids. Determination has been made based on the survey spectra of analyzed samples using CasaXps software.

	**MWCNT**	**MWCNT oxidized**	**MWCNT-BSA**	**MWCNT-BSA-QD**
**O**	6.5	36.1	22.2	24.8
**C**	93.5	50.3	66.7	66.4
**N**	0.0	0.0	8.8	5.2
**S**	0.0	0.0	0.0	1.2
**Cd**	0.0	0.0	0.0	0.4
**Te**	0.0	0.0	0.0	0.3
**Cu**	0.0	0.0	0.4	0.5
**Fe**	0.0	2.6	1.9	1.2
**Co**	0.0	4.8	0.0	0.0
**Ni**	0.0	3.4	0.0	0.0
**Na**	0.0	2.7	0.0	0.0

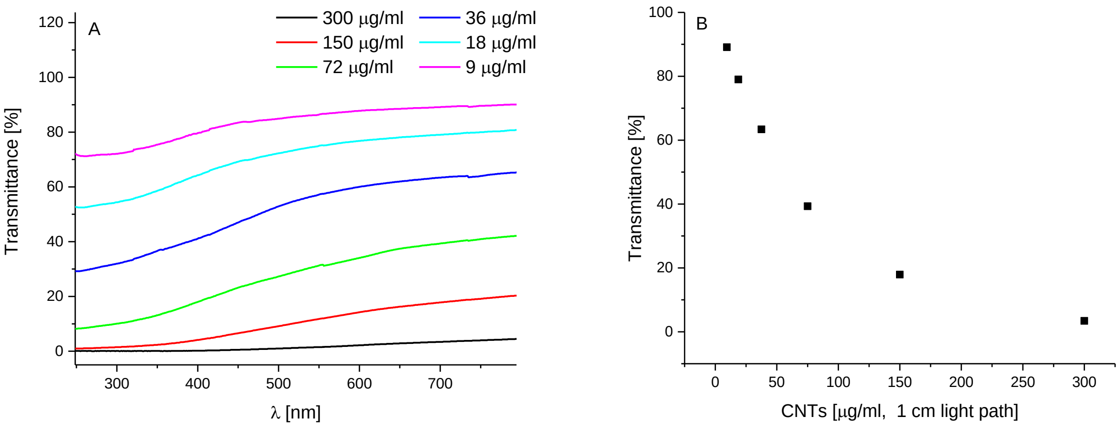

Figure S1. Transmittance changes in the dependence on the CNT concentration. (A) Original transmittance scan recorded for given CNT suspensions and (B) change in the transmittance at 650 nm as the function of CNT concentration.

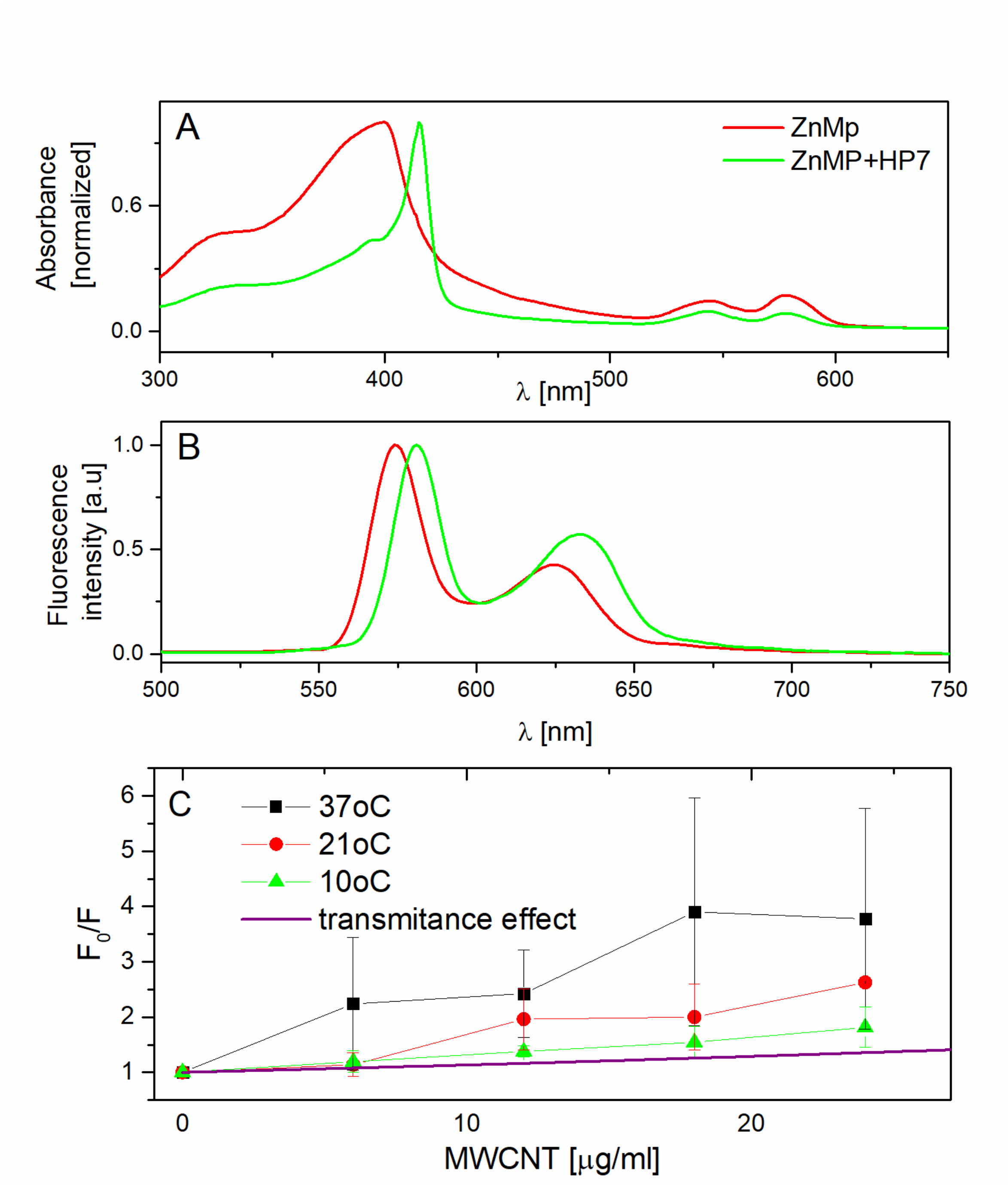

Figure S2. Absorption (A) and emission (B) spectra of Zn-mesoprohyrine, free (red) or bound to HP7 (green) (1:2, protein:ligand ratio) as well as the titration of HP7-ZnMP with MWCNT at different temperatures (C). For titration with MWCNTs, a protein concentration was 2 µM in a final 1 ml of 25 mM Hepes/NaOH pH 7.5, MWCNTs were added in 1-2 µM aliquots from concentrated stocks solution. Absorption and emission spectra normalized to 1 at maximum. Fluorescence was excited at 405 nm. For the titration curves, emission at 630 nm was followed. For the explanation of “transmittance effect” see Figure S2 and main manuscript.

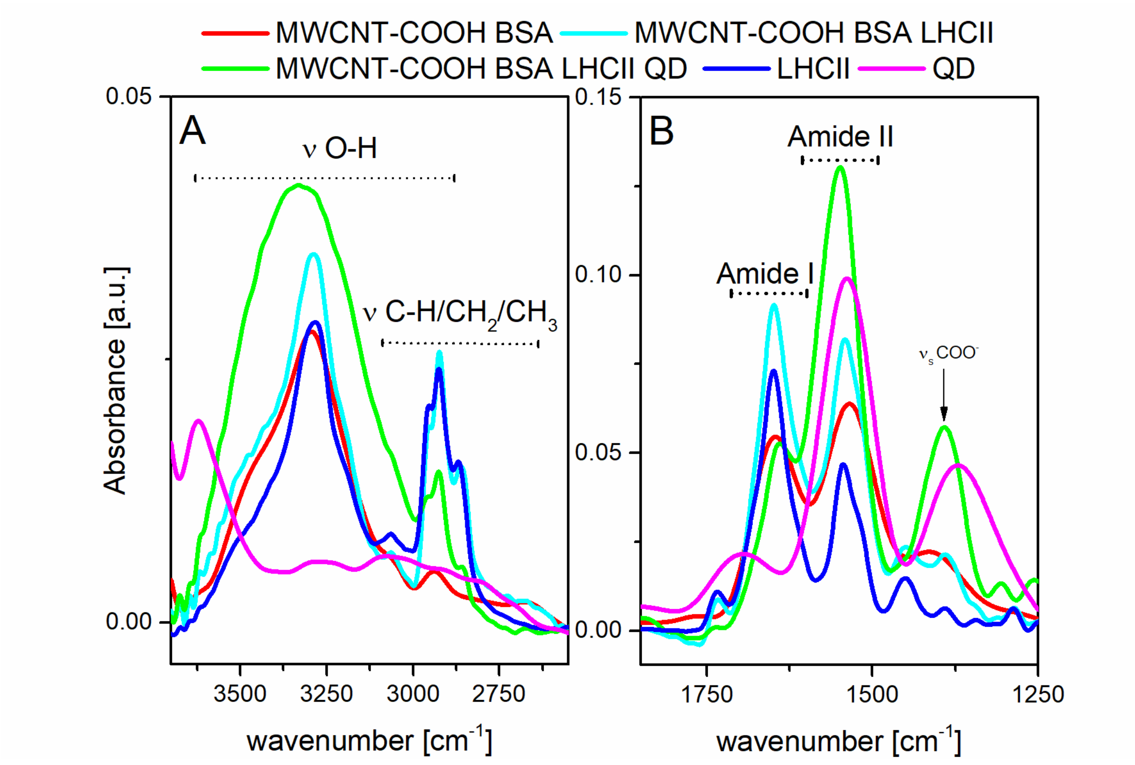

Figure S3. FT-IR analysis of the CNT conjugation process with LHCII. (A) OH and CH vibration range and (B) amide vibration range, recorded for oxidized version of MWCNTs decorated with BSA, BSA-LHCII and BSA-LHCII-QD570. LHCII and QD570 spectra are shown for reference. No normalization was applied.

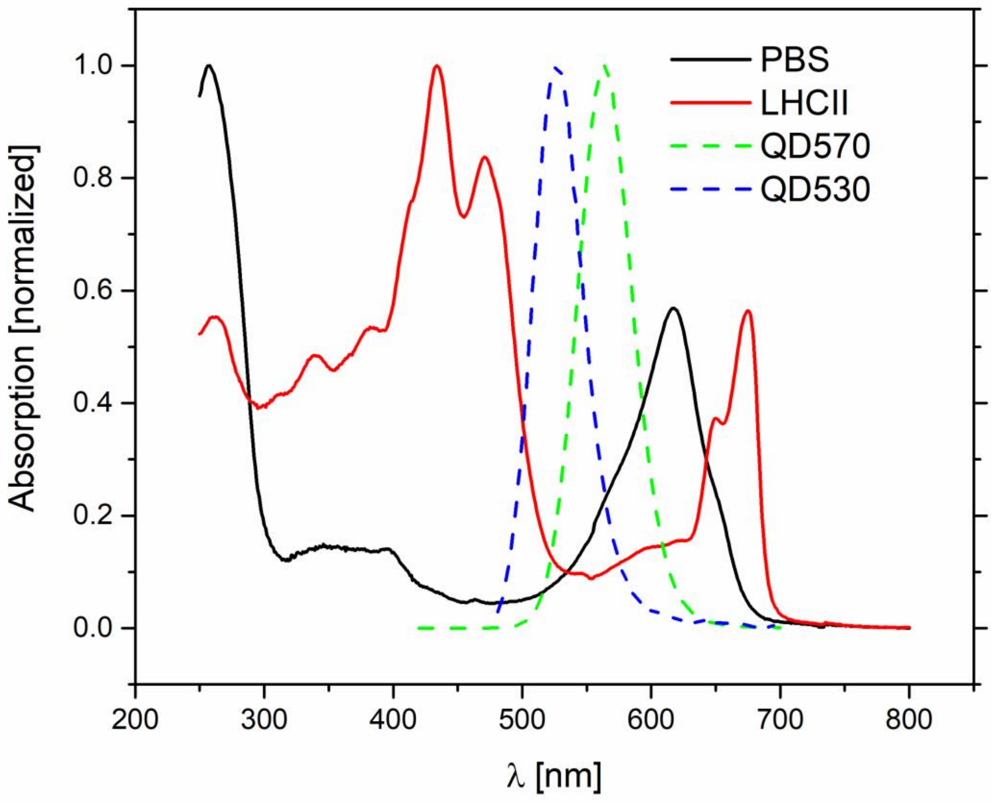

Figure S4. The background for FRET reaction in the studied nanohybrids: emission spectra of donors (QD530 or QD570) and absorption spectra of acceptors (LHCII and PBS).

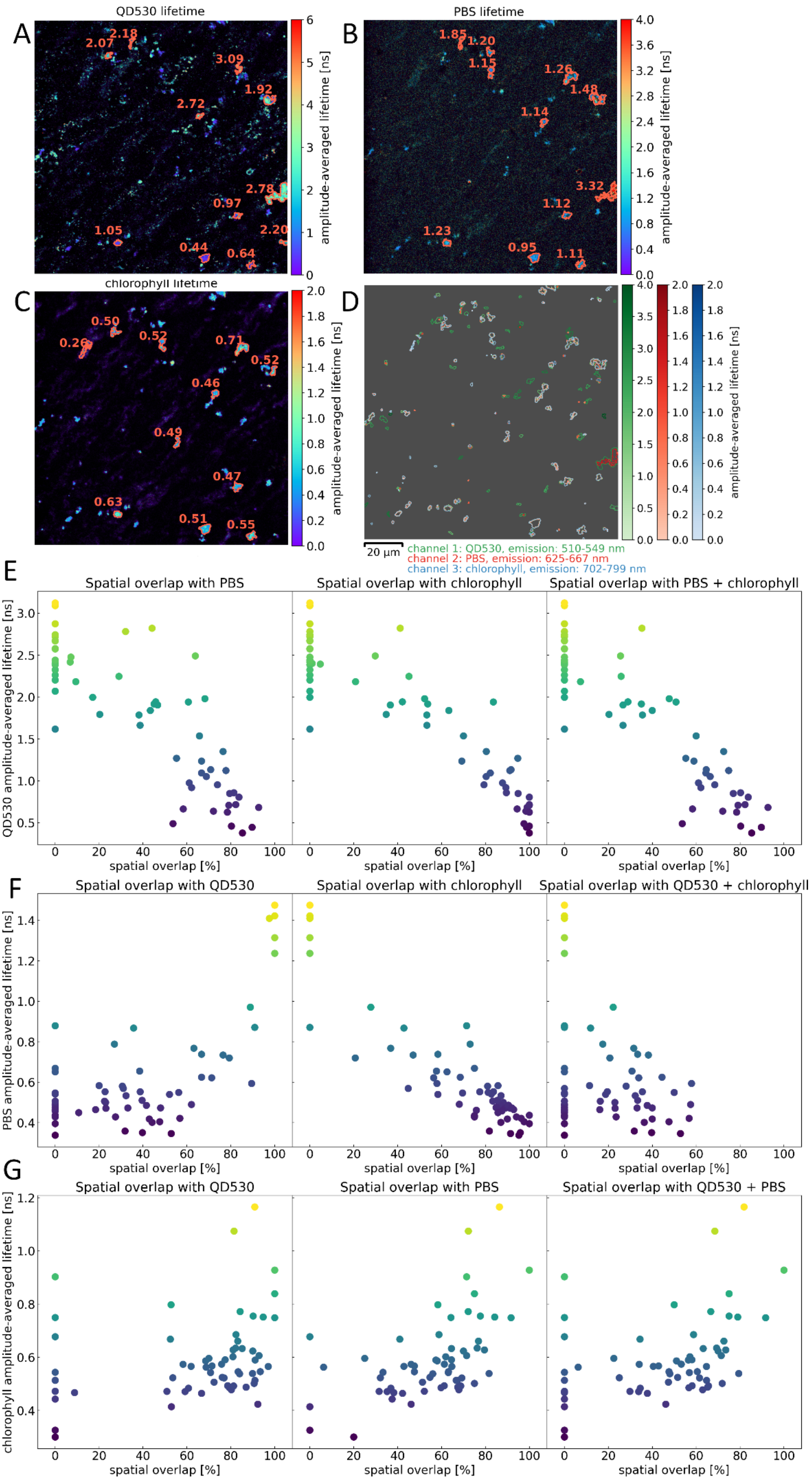
A
QD530 lifetime
2.07
2.18
3.09
1.92
2.72
2.78
0.97
2.20
1.05
0.44
0.64
amplitude-averaged lifetime [ns]
B
PBS lifetime
1.85
1.20
1.15
1.26
1.48
1.14
3.32
1.12
1.23
0.95
1.11
amplitude-averaged lifetime [ns]
C
chlorophyll lifetime
0.50
0.26
0.52
0.71
0.52
0.46
0.49
0.47
0.63
0.51
0.55
amplitude-averaged lifetime [ns]
D
amplitude-averaged lifetime [ns]
20 µm
channel 1: QD530, emission: 510-549 nm
channel 2: PBS, emission: 625-667 nm
channel 3: chlorophyll, emission: 702-799 nm
E
Spatial overlap with PBS
Spatial overlap with chlorophyll
Spatial overlap with PBS + chlorophyll
QD530 amplitude-averaged lifetime [ns]
spatial overlap [%]
F
Spatial overlap with QD530
Spatial overlap with chlorophyll
Spatial overlap with QD530 + chlorophyll
PBS amplitude-averaged lifetime [ns]
spatial overlap [%]
G
Spatial overlap with QD530
Spatial overlap with PBS
Spatial overlap with QD530 + PBS
chlorophyll amplitude-averaged lifetime [ns]
spatial overlap [%]

Figure S5. (on the previous page) The representative example of the FLIM analysis for MWCNT-QD530-PBSr (~300 μg/ml) and thylakoid mixture (100 μg/ml of chlorophyll). (A, B, C) FLIM images of QD530, PBSr, and thylakoid, respectively. Averaged lifetimes [ns] for selected ROIs are indicated. The excitation wavelength was 500 nm, and the emission ranges for each channel listed. (D) The overlap image of ROIs extracted from FLIM images. The contours of the individual ROIs are colored according to their lifetime. (E, F, G) The dependence of the QD530, PBS and chlorophyll lifetime on the spatial overlap with the other components of the FRET system. The points represent individual ROIs, colored according to the lifetime. The dotted line shows the averages from the points in the overlap value ranges spanning 10 percentage points each.

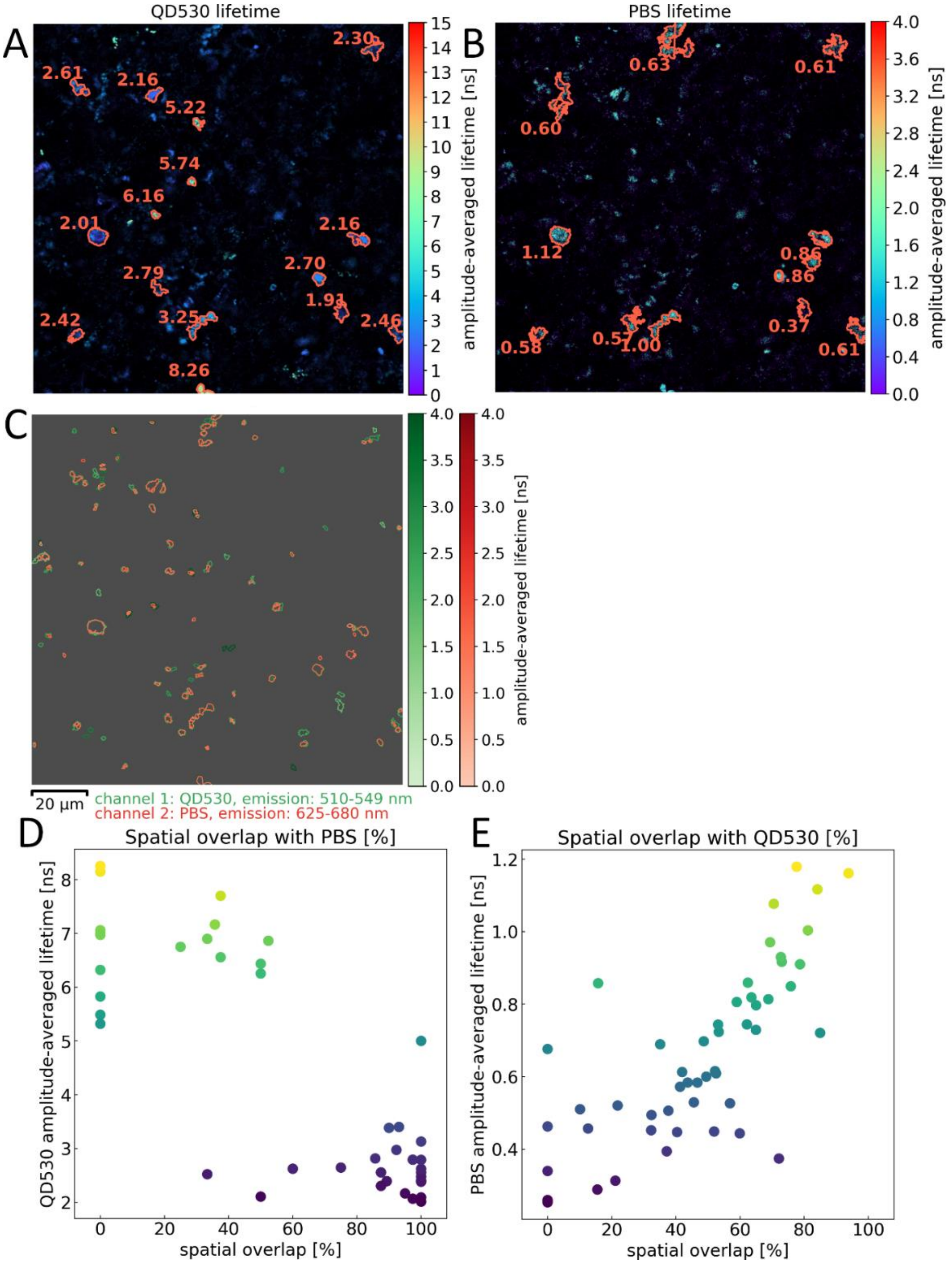

Figure S6. The representative example of the FLIM analysis for MWCNT-QD530-PBSr hybrid (~300 μg/ml). (A, B) FLIM images of QD530 and PBSr. Averaged lifetimes [ns] for selected ROIs are indicated. The excitation wavelength was 500 nm, and the emission ranges for each channel were listed. (C) The

overlap image of ROIs extracted from FLIM images. The contours of the individual ROIs are colored according to their lifetime. (D, E) The dependence of the QD530 and PBS lifetime on the spatial overlap with the other component of the FRET system. The points represent individual ROIs, colored according to the lifetime. The dotted line shows the averages from the points in the overlap value ranges spanning 10 percentage points each.

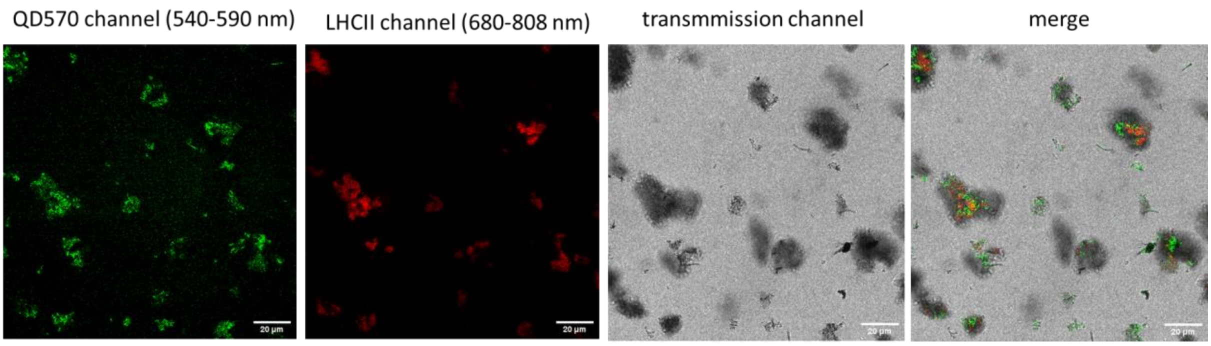

Figure S7. CLSM images of MWCNT-QD570-LHCII hybrids and their mixture with Synechocystis PCC 6803 thylakoids. The excitation wavelength was 470 nm, and the emission ranges for each channel are indicated.

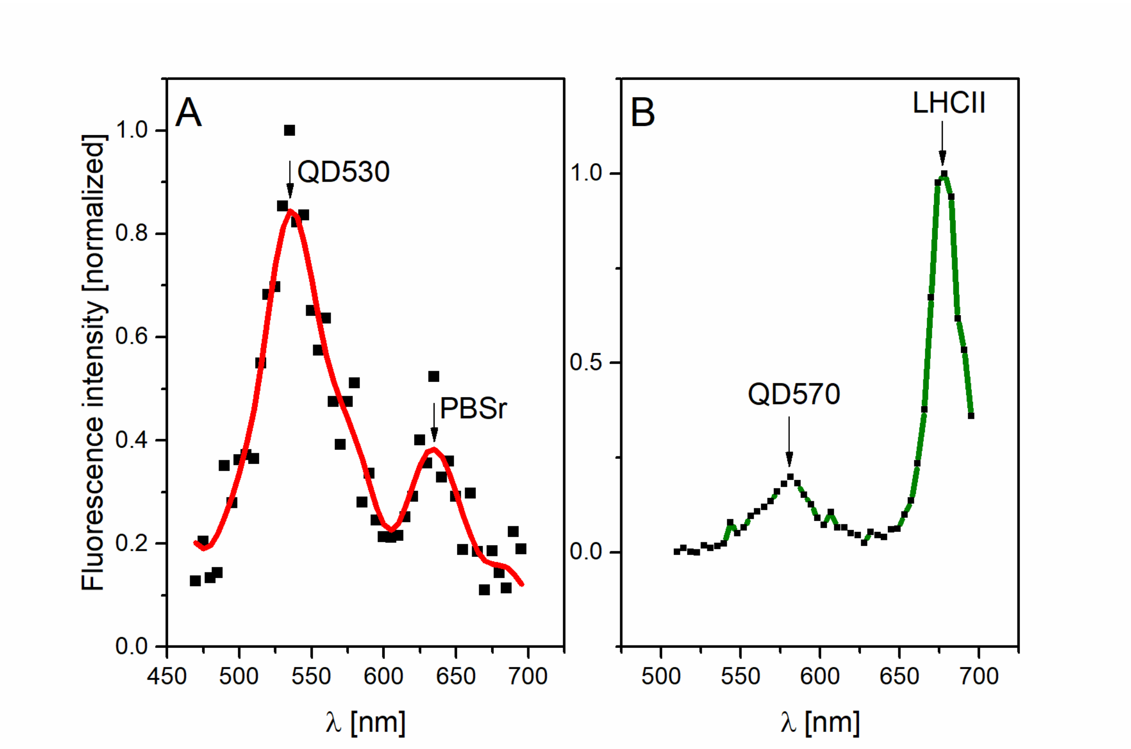

Figure S8. Fluorescence emission spectra recorded with CLSM for selected ROI on MWCNT-BSA-QD530 (A) or MWCNT-BSA-QD570-LHCII (B) preparation. Please note that points represent actual data of the summarized region pixel intensities and the bold line works as an eye-guide only. Emission was excited with 405 nm (A) or 470 nm (B) lase lines. Spectral resolution was set at 3 nm, maximum possible with the system. Arrows indicate position of emission maxima of QD, PBSr and LHCII. Note, the intensity of particular bands do not necessarily represent the amount of fluorophore, as the excitation wavelength may not be optimal for maximum fluorescence.

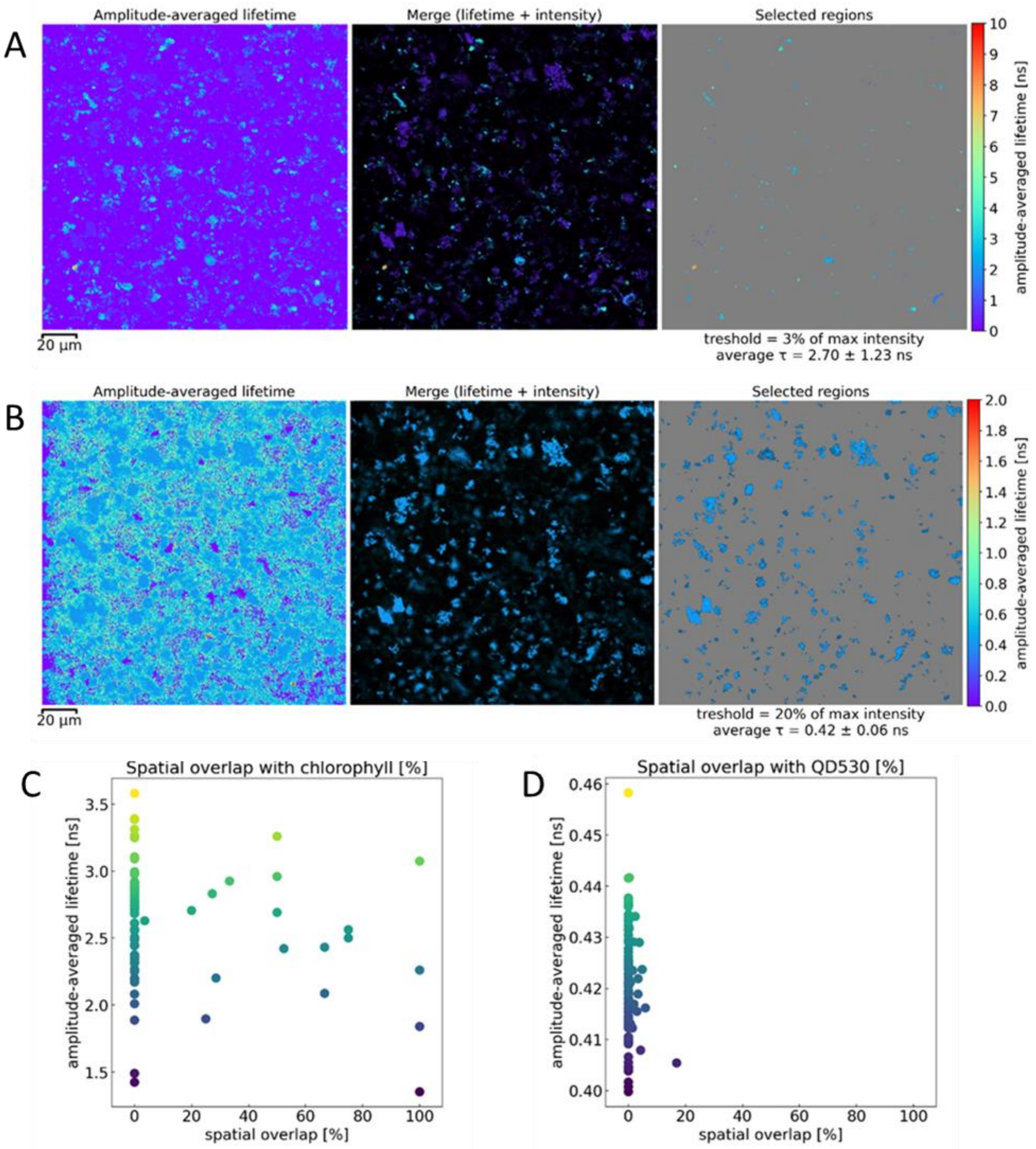

Figure S9. The representative example of the FLIM analysis for MWCNT-QD530 hybrib (~300 µg/ml) mixed with thylakoids. (A) FLIM images of a mixture with QD530 ROIs andalysed and (B), chlorophyll emission ROIs analysis for the same mixture. The excitation wavelength was 500 nm, and the emission ranges were 515-554 and 702-770, respectively. (C, D) The dependence of the QD530 and PBS lifetime on the spatial overlap with the other component of the FRET system. The points represent individual

ROIs, colored according to the lifetime. The dotted line shows the averages from the points in the overlap value ranges spanning 10 percentage points each.

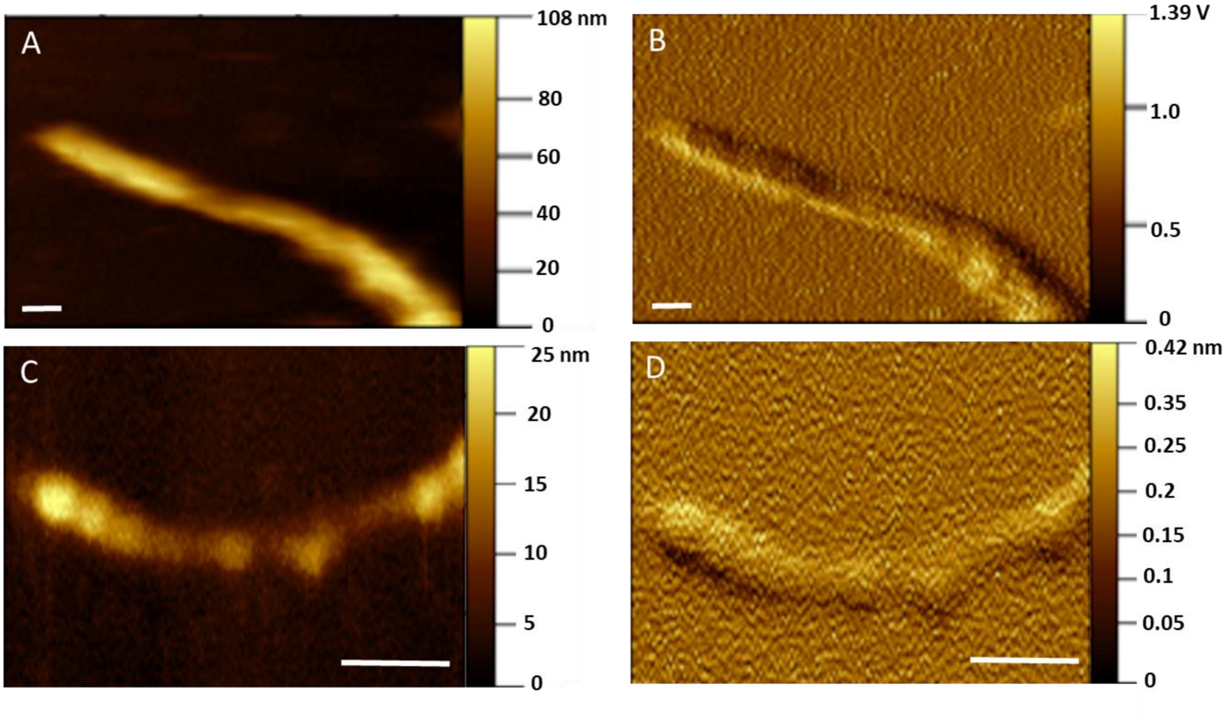

Figure S10. Atomic force microscopy of BSA-coated (A, B) and fully functionalized (C, D) single-walled carbon nanotubes. Images show topography (A, C) and respective deflection image (B, D).

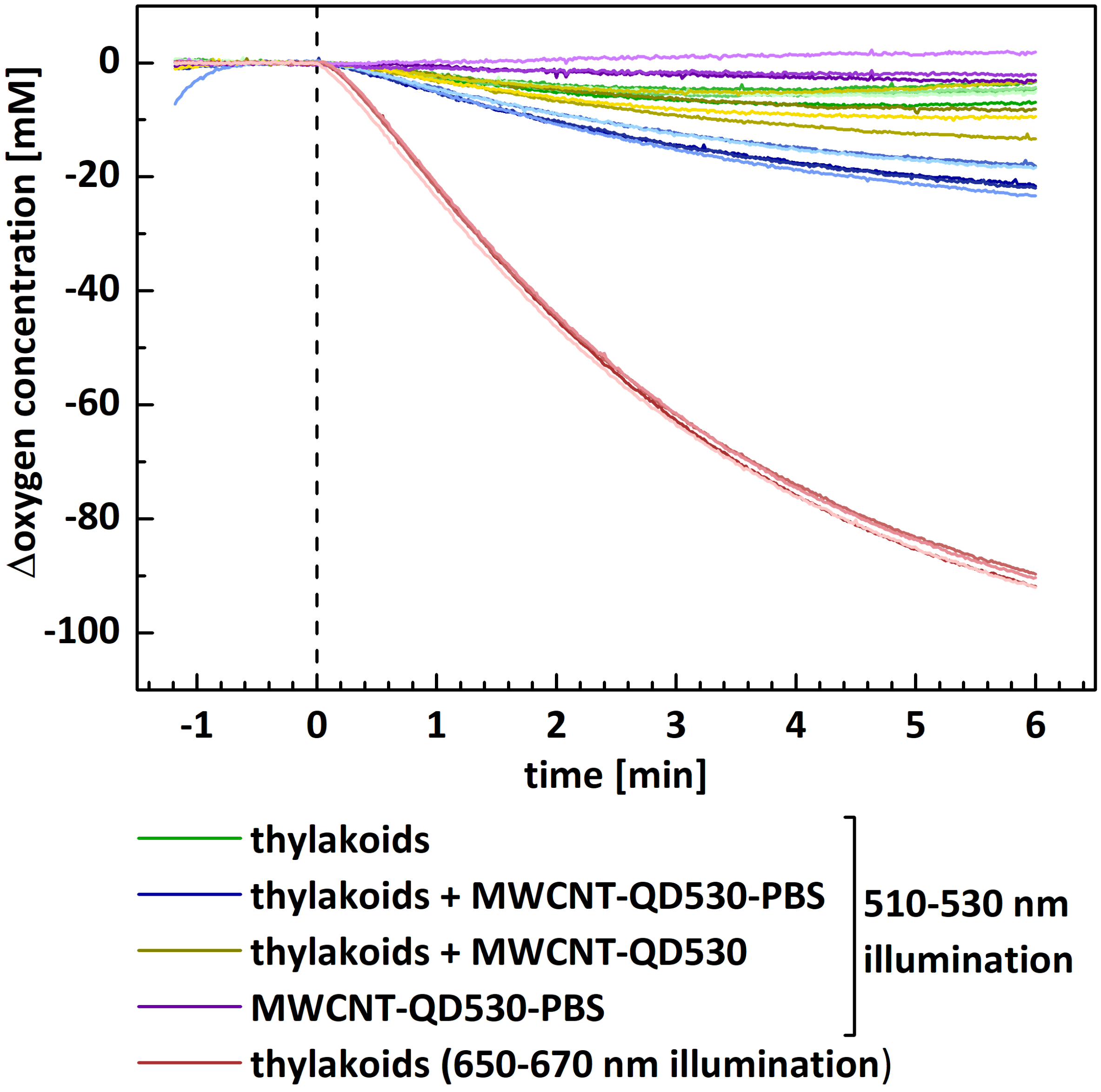

Figure S11. Examples of oxygen consumption, recorded for thylakoids in the absence or presence of MWCNT-QD530-PBSr, illuminated with 510-530 nm light range (bandpass filter,510-530 nm) and the controls for this reaction (MWCNT-QD530-PBSr added to the buffer without thylakoids, and thylakoids illuminated in presence of nanohybrids without PBSr, MWCNT-QD530-PBSr). In comparison, thylakoids illuminated with optimal light range (bandpass filter 650-670 nm). The samples contained the number of thylakoids corresponding to 20 µg/ml chlorophyll and ~ 400 µg/ml nanohybrid in the presence of a redox system (1 mM sodium ascorbate + 0.1 mM DCPIP + 0.5 mM methyl viologen).

Supplementary References

[1] S. Van der Walt, J.L. Schönberger, J. Nunez-Iglesias, F. Boulogne, J.D. Warner, N. Yager, E. Gouillart, T. Yu, scikit-image: image processing in Python, PeerJ, 2 (2014) e453.
